# Towards a replicator dynamics model of age structured populations

**DOI:** 10.1007/s00285-021-01592-4

**Published:** 2021-04-02

**Authors:** K. Argasinski, M. Broom

**Affiliations:** 1 Institute of Mathematics of Polish Academy of Sciences, ul. Śniadeckich 8, 00-656 Warsaw, Poland; 2 Department of Mathematics, University of Sussex, Brighton, BN1 9QH UK; 3 Department of Mathematics, City, University of London, Northampton Square, London, EC1V 0HB UK

## Abstract

We present a new modelling framework combining replicator dynamics, the standard model of frequency dependent selection, with an age-structured population model. The new framework allows for the modelling of populations consisting of competing strategies carried by individuals who change across their life cycle. Firstly the discretization of the McKendrick von Foerster model is derived. We show that the Euler–Lotka equation is satisfied when the new model reaches a steady state (i.e. stable frequencies between the age classes). This discretization consists of unit age classes where the timescale is chosen so that only a fraction of individuals play a single game round. This implies a linear dynamics and individuals not killed during the round are moved to the next age class; linearity means that the system is equivalent to a large Bernadelli–Lewis–Leslie matrix. Then we use the methodology of multipopulation games to derive two, mutually equivalent systems of equations. The first contains equations describing the evolution of the strategy frequencies in the whole population, completed by subsystems of equations describing the evolution of the age structure for each strategy. The second contains equations describing the changes of the general population’s age structure, completed with subsystems of equations describing the selection of the strategies within each age class. We then present the obtained system of replicator dynamics in the form of the mixed ODE-PDE system which is independent of the chosen timescale, and much simpler. The obtained results are illustrated by the example of the sex ratio model which shows that when different mortalities of the sexes are assumed, the sex ratio of 0.5 is obtained but that Fisher’s mechanism, driven by the reproductive value of the different sexes, is not in equilibrium.

## Introduction

Among the most important approaches to the modelling of evolutionary processes are life history optimization and evolutionary games. Classical life history theory (Stearns 1992; Roff 1992) relies on optimization models, where there are no interactions among individuals and no density dependence:

*“Life history evolution usually ignores density and frequency dependence. The justification is convenience, not logic, or realism”* (Stearns 1992).

On the other hand, in classical game theoretic models there is no age or stage structure. Payoffs describe the averaged lifetime activity of an individual, which can be found for example in Cressman (1992):

*“...an individual’s strategy is fixed over its lifetime or, alternatively, the life history of an individual is its strategy.”*

Thus the synthesis of these perspectives can be very fruitful for theoretical insight (McNamara 2013). Methods used in life history optimization are closely related to classical demographic methods such as Bernadelli–Lewis–Leslie matrices (Caswell 2001). However, how to construct a general description of the relationships between demographic structure and population dynamics is still an unsolved problem (Caswell 2011). More precise than matrix models are continuous approaches arising from Lotka’s renewal equation (Lotka 1911, Diekmann et al. 2020a, b) and the McKendrick von Foerster model (McKendrick 1926). The combination of demography with a game theoretic perspective focused on frequency dependent selection, advocated by McNamara (2013), can be very useful since demographers are interested in the patterns produced by heterogeneity in the populations (Vaupel et al. 1979; Vaupel and Yashin 1983; Hougaard 1984; Vaupel and Yashin 1985). The game theoretic structure can explain the mechanisms shaping those patterns. The first papers combining both approaches are Garay et al. (2016) devoted to the particular biological problem of sib cannibalism, Li et al. (2015) and Lessard and Soares (2018) containing the approach incorporating age structure into a matrix game. These results show that after introduction of the age structure, matrix notation becomes very complicated and makes analysis difficult even in the case of two competing strategies and few age classes. In addition, previous works do not study the interplay between game dynamics and demographic structure in detail, assuming a fixed demographic structure. However, the game interactions described by demographic payoffs should affect the demographic structures of subpopulations of carriers of different strategies. In addition Li et al. (2015) assumes that payoffs are described by a standard payoff matrix, thus the same actions performed in different ages/stages will generate the same payoffs. However, we can expect that outcomes of individual actions may vary for different ages due to different experiences and physical condition of the playing individuals.

Another problem is that game theoretic models operate in abstract terms of costs and benefits measured in units of fitness mostly without deeper insight into their meaning or interpretation. This problem was analyzed in Argasinski and Broom (2012) where relationships between classical demography and evolutionary games are described in detail. This approach was later clarified in Argasinski and Broom (2018a, 2018b) by definition of the vital rates (birth and death rates) as the product of the interaction rates, describing the distribution of interactions (game rounds) in time and demographic game payoffs describing the number of offspring and the probability of death during a single interaction. The main conclusion there is that instead of excess above average fitness, models should be described explicitly by mortality and fertility, which are basic opposite forces shaping population dynamics (Doebeli et al. 2017). These results are significant progress in ecological realism, emphasizing the role of background mortality and fertility or the turnover of individuals (Argasinski and Kozłowski 2008). However, that approach is still very primitive. Mortality is described as an exponential decay of the population, which implies that the length of an individual’s lifetime is potentially unbounded, and there is no aging and no age specific payoffs. The goal of this paper is to fill this gap and develop a mathematical structure combining selection of individual strategies with an age structured population which will allow us to overcome the problems arising from increasing complexity of the models shown in Li et al. (2015) and simplifications ignoring the age dependence of payoffs resulting from certain actions and feedbacks driving the interplay between game dynamics and demography (the fixed age structure assumption). For practical reasons we will develop a high dimensional ODE system consisting of relatively simple equations, which can be generated by a simple loop and solved in every popular numerical platform.

**Table 1:** List of important symbols

*n* -population size	
$n_{j}$-number of individuals carrying the *j*th strategy	
$\tau$-interaction rate	
$f_{j}$ ($f_{j}^{i}$)-fertility payoff of the *j*th strategy (of the *j*th strategy at age *i* )	
$s_{j}$ ($s_{j}^{i}=1-\tau d_{j}^{i}$)-survival payoff of the *j*th strategy (of the *j*th strategy at age *i* )	
$\bar{f}_{j}$, $\bar{f}^{i}$, $\bar{f}$-average fertility for the *j*th strategy, *i*th age class, whole population	
$\bar{s}_{j}$, $\bar{s}^{i}$, $\bar{s}$-average survival for *j*th strategy, *i*th age class, whole population	
$\bar{r}=\tau \tilde{r}$-Malthusian parameter, product of the interaction rate and the game payoffs	
$m+1$-number of age classes	
*w*-number of strategies	
*K*-carrying capacity, maximal population load	
$a_{j}^{i}$-frequency of individuals at age *i* among *j*-strategists	
$p_{j}$-frequency of *j*-strategists in the population	
$a^{i}$-proportion of individuals in the *i*th age class	
$p_{j}^{i}$-frequency of *j*-strategists in the *i*th age class	
$P_{j}$-sex ratio strategy (fraction of males in the brood of the female)	
*x* ($x_{j}$)-number of females (carrying the *j*th strategy)	
*y* ($y_{j}$)-number of males (carrying the *j*th strategy)	
$G_{j}=\left( x_{j}+y_{j}\right) /\sum _{l=1}^{w}\left( x_{l}+y_{l}\right)$-frequency of the *j*th strategy gene	
$P=y/(x+y)$ -secondary sex ratio (proportion of males)	
$\bar{P}_{pr}$-primary sex ratio (average strategy of females)	
$S_{j}^{f}=\sum _{l=c}^{d}a_{j}^{l}(1-M_{j}^{l})$-proportion of active females among the *j*th strategy carriers	
$S_{j}^{m}=\sum _{l=a}^{b}a_{j}^{l}M_{j}^{l}$-proportion of active males among the *j*th strategy carriers	
$M_{j}$ ($M_{j}^{i}$)-sex ratio of the population of the *j*th strategy carriers (of *j*th strategy carriers at age *i*)	
*k*-number of offspring in the brood of a female	
$M_{j}^{op}=S_{j}^{m}/\left( S_{j}^{m}+S_{j}^{f}\right)$-operational sex ratio of *j*th strategists	
$P_{op}=\bar{S}^{m}/\left( \bar{S}^{m}+\bar{S}^{f}\right)$-operational sex ratio in the population	

### The classical approach to evolutionary games and replicator dynamics

In the following subsections, we describe the state of the art in relation to our problem. A list of existing (and indeed new, see later) parameters are described in Table 1. Traditionally, in evolutionary game theory the payoff obtained by the *j*th strategy is proportional to its Malthusian growth rate $r_{j}$ and the dynamics of selection of strategies is described by the *replicator dynamics* (Maynard Smith 1982; Cressman 1992; Hofbauer and Sigmund 1988, 1998; Weibull 1995; Nowak 2006; Broom and Rychtár 2013; McNamara and Leimar 2020). We can derive this by rescaling the Malthusian equations for competing strategies $\dot{n} _{j}=n_{j}r_{j}$ to relative frequencies $q_{j}=n_{j}/n$ (where $n=\sum _{j=1}^{w}n_{j}$ and *w* is the number of strategies), which leads to

1 $$\begin{aligned} \dot{q}_{j}=q_{j}(r_{j}-\bar{r}) \end{aligned}$$

where $\bar{r}=\sum _{j=1}^{w}q_{j}r_{j}$ is the average payoff in the population. However, instead of the Malthusian parameter describing the payoff we can explicitly consider the individual fertility $f_{j}$ and mortality $d_{j}$ of a *j*-strategist. The explicit distinction between fertility and mortality was proposed also by Doebeli et al. (2017) as the cornerstone of a mechanistic model of natural selection. Note that in real life organisms are involved in different types of interactions with others or elements of the environment. Game theoretic models are focused on the outcomes of the particular interactions (such as fights as in the Hawk Dove game) responsible for selection of the analyzed trait or type of behaviour. These can be described by average demographic outcomes per interaction $f_{j}$ and $d_{j}$ and these focal interactions will occur at the rate $\tau _{f}$. Other interactions, not related to the analyzed trait, can be described by average fertility $f_{b}$ and mortality $d_{b}$, occurring at rate $\tau _{b}$.

Products of interaction rates and demographic payoffs will constitute the respective *vital rates*: game fertility rate $\tau _{f}f_{j}$ and mortality rate $\tau _{f}d_{j}$, background fertility rate $\tau _{b}f_{b}$ and mortality rate $\tau _{b}d_{b}$. Later the focal game interaction rate $\tau _{f}$ can be set to 1 by timescale adjustment and the background fertility and mortality rates become $\Phi =\tau _{b}f_{b}/\tau _{f}$ and $\Psi =\tau _{b}d_{b}/\tau _{f}$. In addition we can add density dependent juvenile recruitment survival (Argasinski and Kozłowski 2008; Argasinski and Broom 2012, 2018a, b). To do this we should multiply fertilities by the logistic suppression coefficient $(1-n/K)$ (where the carrying capacity *K* is interpreted as the maximal environmental load, Hui 2006). Since fertilities but not mortalities are so scaled, the turnover of generations will not be suppressed at the equilibrium as it is in the classical logistic model (which leads to an immortal and childless population at equilibrium *K*). This gives the following variant of the replicator equations:

2 $$\begin{aligned} \dot{q}_{j}= & {} q_{j}\left( (f_{j}-\bar{f})\left( 1-\frac{n}{K}\right) -(d_{j}-\bar{ d})\right) , \end{aligned}$$

3 $$\begin{aligned} \dot{n}= & {} n\left( \left[ \bar{f}+\Phi \right] \left( 1-\frac{n}{K}\right) -\bar{d} -\Psi \right) , \end{aligned}$$

where $\bar{f}=\sum _{j=1}^{w}q_{j}f_{j}$ and $\bar{d} =\sum _{j=1}^{w}q_{j}d_{j}$, the details of which appear in Argasinski and Broom (2012), Argasinski and Broom (2018a, 2018b).

It was shown (Argasinski 2006) that every single population system described by the replicator Eq. (1) can be divided into the product of subsystems describing the dynamics in arbitrary chosen disjoint subpopulations (described by a frequencies $q_{j}^{i}=n_{j}^{i}/n_{j}$, where $n_{j}=\sum _{i}n_{j}^{i}$, for the *j*-th subpopulation) and an additional system describing the dynamics of proportions between those subpopulations $p_{j}=n_{j}/\sum _{z}n_{z}$. This is useful when indiviuals differ not only by strategies but also by another second trait such as sex, age or developmental stage. Then, for example, we can decompose the population into subpopulations of carriers of the same strategy and describe the dynamics of the second trait among them. Then the dynamics in each subpopulation will have the form (1) and will depend on the excess of the strategy payoff from the average payoff in this subpopulation. Therefore, the same operation can be carried out for Eq. (2), and we obtain the system:

4 $$\begin{aligned} \dot{q}_{j}^{i}= & {} q_{j}^{i}\left( \left( f_{j}^{i}-\bar{f}_{j}\right) \left( 1-\frac{n}{K}\right) -\big (d_{j}^{i}-\bar{d}_{j}\big )\right) , \end{aligned}$$

5 $$\begin{aligned} \dot{p}_{j}= & {} p_{j}\left( \left( \bar{f}_{j}-\bar{f}\right) \left( 1-\frac{n }{K}\right) -\big (\bar{d}_{j}-\bar{d}\big )\right) , \end{aligned}$$

6 $$\begin{aligned} \dot{n}= & {} n(\left[ \bar{f}+\Phi \right] \left( 1-\frac{n}{K}\right) -\bar{d} -\Psi ), \end{aligned}$$

where $f_{j}^{i}$ and $d_{j}^{i}$ are the fertility and mortality, respectively, of the *i*- th type (such as age or sex) in the subpopulation of the *j*-th strategy carriers, $\bar{f}_{j}= \sum _{i=1}^{w}q_{j}^{i}f_{j}^{i}$ and $\bar{d}_{j}= \sum _{i=1}^{w}q_{j}^{i}d_{j}^{i}$ are the mean fertility and mortality, respectively, in the subpopulation of the *j*-th strategy carriers and $\bar{ f}$ and $\bar{d}$ are the respective values in the global population.

Note that we can decompose the initial population with respect to the second trait and describe the dynamics of strategic composition among individuals in the same age or sex class. Then the equations will describe variables $$ q_{j}^{i}$$, $$p_{j}$$ and *n*.

### The classical approach to the modelling of age structured populations

Now we focus on age structured models (age classes will be indexed by superscripts). The classical approach to the modelling of age structured populations is related to Bernadelli–Lewis–Leslie matrices (Bernadelli 1941; Lewis 1942; Leslie 1945; Charlesworth 1994; Caswell 2001), following the matrix equation:

7 $$\begin{aligned} \left[ \begin{array}{c} n^{0} \\ n^{1} \\ \ldots \\ n^{m} \end{array} \right] _{t+1}=\left[ \begin{array}{cccc} f^{0} &{} f^{1} &{} \ldots &{} f^{m} \\ s^{0} &{} 0 &{} 0 &{} 0 \\ 0 &{} \ldots &{} 0 &{} 0 \\ 0 &{} 0 &{} s^{m-1} &{} 0 \end{array} \right] \left[ \begin{array}{c} n^{0} \\ n^{1} \\ \ldots \\ n^{m} \end{array} \right] _{t}, \end{aligned}$$

where there are $m+1$ age classes, $n^{i}$ is the size of the *i*th age class and $f^{i}$ is fertility and $s^{i}$ is survival, respectively, in this class. Thus $n^{0}(t+1)=\sum _{i}n^{i}(t)f^{i}$ and the transition between subsequent age classes is $n^{i}(t+1)=s^{i-1}n^{i-1}(t)$. When the time unit equals the time step between age classes the above system is a good model of age structure. This age-structured growth model suggests a steady-state, or stable, age-structure and growth rate. The growth rate can be calculated from the characteristic polynomial of the Bernadelli–Lewis–Leslie Matrix called the Euler–Lotka equation (Caswell 2001):

8 $$\begin{aligned} f^{0}+\sum \limits _{i=1}^{m}e^{-ir}f^{i}\prod \limits _{z=0}^{i-1}s^{z}=1, \end{aligned}$$

where *r* is the intrinsic growth rate of the population and $\prod _{z=0}^{i-1}s^{z}$ describes survival to age *i*. We note here that in reality *r* will not be an independent parameter, and moreover will change in time as the distributions of the sizes of age classes change. An equilibrium distribution over the age classes in turn will allow us to define *r* in terms of the other model parameters. A simple ODE generalization of this system with continuous time but discrete age structure can be obtained by application of the delayed differential equations (Caswell 2001) where survival rates may describe aggregated exponential survival between respective age classes (Diekmann et al. 2017). However this approach may not work if the mortality function depends on the actual population state (as in game theory). Here the mortality rate may be unknown since it will depend on the trajectory of the dynamics during the age class. Then we can consider the continuous time limit of an infinite number of infinitely small age classes where population structure becomes a function *n*(*t*, *l*) of time *t* and continuous age *l* describing a moment in the lifetime of an individual. Then we can imagine the Taylor expansion analogous to the transition equation describing a small time step *dt* leading to ageing *dl*

9 $$\begin{aligned} n(t+dt,l+dl)= & {} n(t,l)+\frac{\partial n}{\partial t}dt+\frac{\partial n}{\partial l} dl=s(l)n(t,l)\nonumber \\= & {} \left( 1-\tau d(l)dt\right) n(t,l), \end{aligned}$$

where $\tau d(l)$ is the continuous time mortality rate (at age *l*) similarly to the game models but without the distinction between the focal game and the background interactions. Since $dl=dt$ we obtain the McKendrick von Foerster equation

10 $$\begin{aligned} \frac{\partial n(t,l)}{\partial t}+\frac{\partial n(t,l)}{\partial l}=-\tau d(l)n(t,l), \end{aligned}$$

which should be completed by boundary conditions $n(t,0)=\int _{0}^{\infty }n(t,l)\tau f(l)dl$ and initial age distribution *n*(0, *l*).

## The paper structure

In this paper we derive the discretization of the McKendrick von Foerster model allowing for the derivation of frequency dependent models. This is motivated by the fact that the discretized approach can be easily numerically solved by basic ODE solvers from popular numerical platforms. Thus the developed methodology does not need advanced knowledge in numerical analysis. Another advantage is that it will be compatible with standard game theoretic notation based on matrix games. Using the derived discretization we build two approaches to modelling selection among competing strategies with life cycles in an asexual population. One is focused on the impact of age structures of strategies on selection, while the second shows the impact of selection dynamics on the age structure of the whole population. The models obtained are generalized to mixed PDE-ODE models with continuous non-discretized age structures to outline the direction of future development. This framework is illustrated by a sex ratio example combining the two approaches, allowing us to model the sexually reproducing population. Our intention is to build a simple ready to use modelling methodology which can be extended in the future. However, we believe that even after clarification of the PDE based approach and development of simple solvers of coupled integro-differential PDE-ODE systems, our approach will still be useful for practical reasons arising from the simplicity of the methods based on matrix payoffs, which are much simpler to derive than continuous payoff functions. Thus it can be, for example, easily used for building initial toy models.

## Results

### Presenting the McKendrick von Foerster model as a system of ODE’s

In this section we will build the submodel describing the age structure dynamics of a subpopulation of carriers of some strategy competing with other strategies. Demographic vital rates will be outcomes of interactions between carriers of different strategies, interpreted as rounds of evolutionary games as in Argasinski and Broom (2018a). Thus as in replicator dynamics models we have the state of the population described by strategy frequencies $p_{j}$ but for each strategy subpopulation we have a respective age structure described by parameters $a_{j}^{i}=n_{j}^{i}/n_{j}$ (frequencies of individuals of age *i* among *j*-th strategy carriers). Demographic payoffs determining the vital rates will depend not only on the strategy frequencies $p=[p_{1},\ldots ,p_{w}]$ as in the classical replicator models but also on the age of the opponents, thus the set of vectors of the age structures for all strategies $a=[a_{1},\ldots ,a_{w}]$ (where $a_{j}=[a_{j}^{0},\ldots ,a_{j}^{m}]$ describes the age structure of the *j*-th strategy carriers subpopulation) should be another argument of the payoff functions.

A major technical difference between the McKendrick von Foerster model and replicator dynamics is that the first is a PDE (or system of PDE’s as for example in Rundnicki and Mackey 1994) and the second is a system of ODE’s. The simple combination of both approaches will lead to a mathematically elegant but technically intractable system due to the lack of a general theory for mixed PDE-ODE systems and software for numerical computation. This methodology should be developed in the future, however before that, we need a useful approach based on existing solutions. To solve this problem we can approximate the continuous system by a large number of ODE’s describing unit interval age classes consisting of all individuals of age from *a* to $$ a+1$$. The discrete structure will allow us to use standard matrix payoff functions. The chosen time unit should be as long as possible to reduce the number of equations. Since we want to model frequency dependent selection, the mortality and fertility payoffs will depend on the trajectory of the population state. Therefore we cannot use simplified delayed differential equations since we do not know the trajectories during the time delay interval. Instead we can assume that the unit of a timescale described by interaction rate $$\tau $$ is short enough that the changes of the population state are small enough with respect to the population size (e.g. 50 births in a population of 30000), that the resulting changes of frequency dependent birth and death rates will be negligible.

**Fig. 1:**
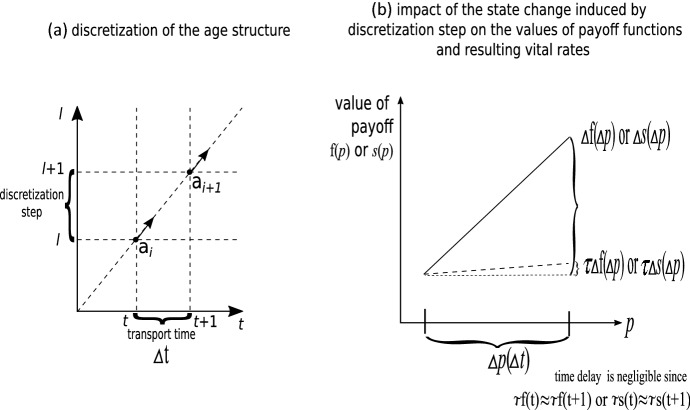
Schematic presentation of the discretization of the continuous age dynamics. The assumed unit time step between age classes is associated with a change of the population state, which may induce change of the frequency dependent payoffs. However, while the resulting changes of the vital rates are negligible, values of payoffs can be approximated by their initial values at the beginning of the transition between age classes

Following “Appendix A” we see that Eq. (10) can be discretized and approximated by the replicator dynamics (see Fig. 1 for the intuitive presentation of the discretization scheme for frequency dependent vital rates). In particular for the *j*th strategy we describe the system in frequencies $a_{j}^{i}=n_{j}^{i}/\sum _{z=0}^{m}n_{j}^{z}$ and a scaling parameter *n*. Assume that $\tilde{r}_{j}^{i}(t)=f_{j}^{i}(p(t),a(t))\left( 1-\dfrac{n}{K}\right) -d_{j}^{i}(p(t),a(t))$ is the game payoff component of the growth rate (then $r_{j}^{i}(t)=\tau \tilde{r}_{j}^{i}(t)$) and $\tilde{r }_{j}(t)=\sum _{i}a_{j}^{i}\tilde{r}_{j}^{i}(t)$ is the respective averaged value. If the growth rates $\tau \tilde{r}_{j}(t)$ are nearly constant, then for the chosen timescale described by interaction rate $\tau$ changes of the strategy frequencies during a single time unit are $\Delta p_{j}=\dfrac{ \tau }{(1+\tau \tilde{r}(t))}p_{j}(t)\left( \tilde{r}_{j}(t)-\tilde{r} (t)\right)$ (where $\tilde{r}(t)=\sum _{j}p_{j}\tilde{r}_{j}(t)$), thus they are sublinear. Here $\tau$ should be as big as possible to minimize the number of equations, but small enough that payoff function arguments $\Delta p_{j}$ (and similarly others) should change their values only slightly (i.e. $\Delta f_{j}^{i}=f_{j}^{i}(p(t)+\Delta p)-f_{j}^{i}(p(t))$ and $\Delta d_{j}^{i}=d_{j}^{i}(p(t)+\Delta p)-d_{j}^{i}(p(t))$ are small enough, but not necessarily infinitesimal), so that the resulting changes of $\tau \Delta f_{j}^{i}$ and $\tau \Delta d_{j}^{i}$ are negligible. Then the discretization is acceptable and we obtain:

11 $$\begin{aligned} \dot{a}_{j}^{i}= & {} a_{j}^{i-1}s_{j}^{i-1}(p,a)-a_{j}^{i}\left( \bar{r} _{j}(p,a,n)+1\right) i=1,\ldots ,m, \end{aligned}$$

12 $$\begin{aligned} \dot{n}_{j}= & {} n_{j}\bar{r}_{j}(p,a,n), \end{aligned}$$

where $a_{j}^{0}=1-\sum _{i=1}^{m}a_{j}^{i}$ and the Malthusian parameter describing the growth of the *j*th strategy is

13 $$\begin{aligned} \bar{r}_{j}(p,a,n)=\sum _{i=0}^{m}a_{j}^{i}\left( \tau f_{j}^{i}(p,a)\left( 1- \dfrac{n}{K}\right) +s_{j}^{i}(p,a)\right) -1. \end{aligned}$$

It is important that age class survival $$s_{j}^{i}(p,a)=(1-\tau d_{j}^{i}(p,a))$$ describes aggregated outcomes of the game rounds occurring during a time unit. Therefore it is distinct from the survival probability of a single round $$1-d_{j}^{i}(p,a)$$ which should be used in trade-off functions when only survivors of the game round can reproduce (Argasinski and Broom 2012, 2018a, b), leading to fertility $$ (1-d_{j}^{i}(p,a))f_{j}^{i}$$. In addition, due to nearly linear behaviour within a single time unit the system (11,12) is equivalent to the large Leslie matrix (7) with survival $$ s_{j}^{i}(p,a)=(1-\tau d_{j}^{i}(p,a))$$ and then parameter $$\tau $$ describes the fraction of individuals that played the single game round. Parameter $$ \tau $$ always acts as the multiplier of game payoffs $$f_{j}^{i}$$ and $$ d_{j}^{i}$$ (thus the resulting survival rate is $$1-\tau d_{j}^{i}$$). Since in the next sections we will focus on the derivation of the dynamics, where the structure of the vital rates is not so important, for simplicity we can incorporate the interaction rate $$\tau $$ into the birth and death vital rates and skip it in the notation. Therefore, below, $$\tau $$ will be hidden inside functions $$f_{j}^{i}$$ and $$s_{j}^{i}$$ which will be interpreted as the vital rates.

Assume the absence of density dependence. Since the r.h.s. of our system (11) is the negative function of $a_{j}^{i}$, the following attracting nullcline manifold exists (for constant mortalities $s_{j}$ this is an attracting steady state):

14 $$\begin{aligned} \hat{a}_{j}^{i}=\dfrac{\hat{a}_{j}^{0}\prod \limits _{z=0}^{i-1}s_{j}^{z}(p,a) }{\left( \bar{r}_{j}(p.a)+1\right) ^{i}}=\dfrac{\hat{a}_{j}^{0}\prod \limits _{z=0}^{i-1}s_{j}^{z}(p,a)}{\left( \sum _{z=0}^{m}\hat{a} _{j}^{z}\left( f_{j}^{z}(p,a)+s_{j}^{z}(p,a)\right) \right) ^{i}}. \end{aligned}$$

Note that $\hat{a}_{j}^{0}$ will satisfy the general form for $\hat{a} _{j}^{i}$ in Eq. (14). In addition the Euler–Lotka equation is satisfied (for a derivation and proof, see “Appendix B”). In the density dependent case, the age structure attractor (14) will change with the growth of the population. Now we can use the derived submodel for derivation of the full model.

**Fig. 2:**
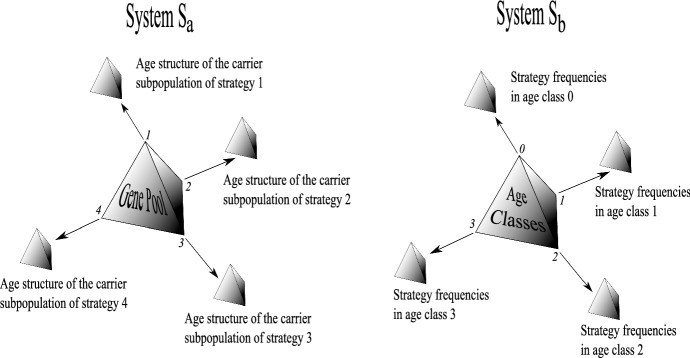
The difference between two alternative formulations of the problem: system **a** describes the evolution of the gene pool according to age structures of carrier subpopulations, system **b** describes the evolution of the global age structure driven by strategy selection in age classes

### The extension to multipopulation replicator dynamics

Now we can incorporate the above model into a multipopulation evolutionary game (Argasinski 2006). Recall that we have *w* strategies and $m+1$ age classes indexed from 0 to *m*. Assume that *p* describes the strategy (phenotype) fraction and *a* describes the frequency of the age class. As before, $f_{j}^{i}$ and $s_{j}^{i}$ describe, respectively, the fertility and survival of the *j*-strategist in age class *i*. Two perspectives are possible (see Fig. 2):

(a) Firstly we consider the impact of the age structure in sub-populations strategically homogenous on selection of the strategies, denoted as system $$ S_{a}$$. This can be described by coordinates:

$a_{j}^{0},\ldots ,a_{j}^{m}$ for $j=1,\ldots ,w$ the age structure of the *j*-strategists

$p_{1},\ldots ,p_{w}$ the strategy frequencies in the whole population,

where $$a_{j}^{i}=n_{j}^{i}/\sum _{z}n_{j}^{z}$$ and $$p_{j}=\sum _{z}n_{j}^{z}/n$$ .

(b) Secondly we consider how selection within each age class affects the overall age structure, denoted as system $S_{b}$. It can be described by coordinates:

$p_{1}^{i},\ldots .,p_{w}^{i}$ for $i=0,\ldots ,m$ strategy frequencies in age class $i$

$a^{0},\ldots ,a^{m}$ the age structure of the population,

where $$p_{j}^{i}=n_{j}^{i}/\sum _{z}n_{z}^{i}$$ and $$a^{i}=\sum _{z}n_{z}^{i}/n$$ .

Thus in both cases we will have a core system describing the whole population (strategic composition in $S_{a}$ and age structure in $S_{b}$) completed by the respective subsystems describing the age structure of the subpopulation of strategy carriers (for $S_{a}$) or the strategic age class composition (for $S_{b}$).

Now we describe the transition of coordinates between the formulations. First we define the auxiliary canonical coordinates without subclasses:

15 $$\begin{aligned} q_{j}^{i}=a^{i}p_{j}^{i}=p_{j}a_{j}^{i}. \end{aligned}$$

Now following Argasinski (2006) we define transitions between systems:

$$S_{a}$$ to $$S_{b}$$:

16 $$\begin{aligned} p^{i}= & {} \left[ p_{1}^{i},\ldots ,p_{w}^{i}\right] =\left[ \dfrac{a_{1}^{i}p_{1} }{\sum _{j=1}^{w}a_{j}^{i}p_{j}},\ldots ,\dfrac{a_{w}^{i}p_{w}}{ \sum _{j=1}^{w}a_{j}^{i}p_{j}}\right] , \end{aligned}$$

17 $$\begin{aligned} a= & {} \left[ a^{0},\ldots ,a^{m}\right] =\left[ \sum _{j=1}^{w}a_{j}^{0}p_{j},\ldots , \sum _{j=1}^{w}a_{j}^{m}p_{j}\right] , \end{aligned}$$

and $$S_{b}$$ to $$S_{a}$$:

18 $$\begin{aligned} a_{j}= & {} \left[ a_{j}^{0},\ldots ,a_{j}^{m}\right] =\left[ \dfrac{a^{0}p_{j}^{0} }{\sum _{i=0}^{m}a^{i}p_{j}^{i}},\ldots ,\dfrac{a^{m}p_{j}^{m}}{ \sum _{i=0}^{m}a^{i}p_{j}^{i}}\right] , \end{aligned}$$

19 $$\begin{aligned} p= & {} \left[ p_{1},\ldots ,p_{w}\right] =\left[ \sum _{i=0}^{m}a^{i}p_{1}^{i},\ldots , \sum _{i=0}^{m}a^{i}p_{w}^{i}\right] . \end{aligned}$$

Now let us derive systems of equations operating in both coordinate systems. In the following we use the within group averaging terms:

$$\begin{aligned} \bar{f}=\sum _{i=0}^{m}a_{j}^{i}f_{j}^{i}, \bar{s}_{j}= \sum _{i=0}^{m}a_{j}^{i}s_{j}^{i}, \bar{s}^{i}= \sum _{j=1}^{w}p_{j}^{i}s_{j}^{i},\ \bar{f}^{i}= \sum _{j=1}^{w}p_{j}^{i}f_{j}^{i}. \end{aligned}$$

We also use two global averages, which can each be written in two ways:

$$\begin{aligned} \bar{f}=\sum _{j=1}^{w}p_{j}\bar{f} _{j}=\sum _{i=0}^{m}a^{i}\bar{f}^{i} \mathrm{and }\,\, \bar{s}=\sum _{j=1}^{w}p_{j}\bar{s} _{j}=\sum _{i=0}^{m}a^{i}\bar{s}^{i}. \end{aligned}$$

For system $S_{a}$ we have the following system of differential equations (see “Appendix C”):

20 $$\begin{aligned} \dot{a}_{j}^{i}= & {} a_{j}^{i-1}s_{j}^{i-1}-a_{j}^{i}\left( \bar{f}_{j}\left( 1-\frac{n}{K}\right) +\bar{s}_{j}\right) \end{aligned}$$

21 $$\begin{aligned} \dot{p}_{j}= & {} p_{j}\left( \left( \bar{f}_{j}-\bar{f}\right) \left( 1-\frac{n }{K}\right) +\left( \bar{s}_{j}-\bar{s}\right) \right) \end{aligned}$$

22 $$\begin{aligned} \dot{n}= & {} n\left( \bar{f}\left( 1-\frac{n}{K}\right) +\bar{s}-1\right) , \end{aligned}$$

giving

23 $$\begin{aligned}&\dot{a}_{j}^{i}=a_{j}^{i-1}s_{j}^{i-1}-a_{j}^{i}\left( \sum _{z=0}^{m}a_{j}^{z}f_{j}^{z}\left( 1-\frac{n}{K}\right) +\sum _{z=0}^{m}a_{j}^{z}s_{j}^{z}\right) , \end{aligned}$$

24 $$\begin{aligned}&\dot{p}_{j}=p_{j}\left( \left( \sum _{i=0}^{m}a_{j}^{i}f_{j}^{i}-\sum _{z=1}^{w}p_{z} \sum _{i=0}^{m}a_{z}^{i}f_{z}^{i}\right) \left( 1-\frac{n}{K}\right) \right. \nonumber \\&\left. \qquad +\left( \sum _{i=0}^{m}a_{j}^{i}s_{j}^{i}-\sum _{z=1}^{w}p_{z} \sum _{i=0}^{m}a_{z}^{i}s_{z}^{i}\right) \right) , \end{aligned}$$

25 $$\begin{aligned}&\dot{n}=n\left( \sum _{j=1}^{w}p_{j}\sum _{i=0}^{m}a_{j}^{i}f_{j}^{i}\left( 1- \frac{n}{K}\right) +\sum _{j=1}^{w}p_{j}\sum _{i=0}^{m}a_{j}^{i}s_{j}^{i}-1\right) . \end{aligned}$$

For system $S_{b}$ we have (see “Appendix D” for a detailed derivation):

26 $$\begin{aligned} \dot{p}_{j}^{0}= & {} \frac{1}{a^{0}}\left( \sum _{i=0}^{m}a^{i}p_{j}^{i}f_{j}^{i}-p_{j}^{0}\bar{f}\right) \left( 1- \dfrac{n}{K}\right) , \end{aligned}$$

27 $$\begin{aligned} \dot{p}_{j}^{i}= & {} \frac{a^{i-1}}{a^{i}}\left( p_{j}^{i-1}s_{j}^{i-1}-p_{j}^{i}\bar{s}^{i-1}\right) , \end{aligned}$$

28 $$\begin{aligned} \dot{a}^{i}= & {} a^{i-1}\bar{s}^{i-1}-a^{i}\left( \bar{f}\left( 1-\dfrac{n}{K} \right) +\bar{s}\right) , \end{aligned}$$

29 $$\begin{aligned} \dot{n}= & {} n\left( \bar{f}\left( 1-\dfrac{n}{K}\right) +\bar{s}-1\right) . \end{aligned}$$

The expanded form of the above system will be

30 $$\begin{aligned}&\dot{p}_{j}^{0}=\frac{1}{a^{0}}\left( \sum _{i=0}^{m}a^{i}p_{j}^{i}f_{j}^{i}-p_{j}^{0}\sum _{i=0}^{m}a^{i} \sum _{z=1}^{w}p_{z}^{i}f_{z}^{i}\right) \left( 1-\dfrac{n}{K}\right) , \end{aligned}$$

31 $$\begin{aligned}&\dot{p}_{j}^{i}=\frac{a^{i-1}}{a^{i}}\left( p_{j}^{i-1}s_{j}^{i-1}-p_{j}^{i}\sum _{z=1}^{w}p_{z}^{i-1}s_{z}^{i-1}\right) , \end{aligned}$$

32 $$\begin{aligned}&\dot{a}^{i}=a^{i-1}\sum _{j=1}^{w}p_{j}^{i-1}s_{j}^{i-1}-a^{i} \sum _{z=0}^{m}a^{z}\left( \sum _{j=1}^{w}p_{j}^{z}f_{j}^{z}\left( 1-\dfrac{n}{ K}\right) +\sum _{j=1}^{w}p_{j}^{z}s_{j}^{z}\right) , \end{aligned}$$

33 $$\begin{aligned}&\dot{n}=n\left( \sum _{i=0}^{m}a^{i}\left( \sum _{j=1}^{w}p_{j}^{i}f_{j}^{i}\left( 1-\dfrac{n}{K}\right) +\sum _{j=1}^{w}p_{j}^{i}s_{j}^{i}\right) -1\right) . \end{aligned}$$

Note that Eq. (33) is equivalent to (25) and in both cases

34 $$\begin{aligned} 1-\dfrac{n}{K}=\dfrac{1-\bar{s}}{\ \bar{f}}\Rightarrow n=K\left( 1-\dfrac{1- \bar{s}}{\ \bar{f}}\right) . \end{aligned}$$

Recall that for simplicity we assumed that functions act as the vital rates with interaction rate $\tau$ hidden inside. When we insert it back it would appear as $\tau f_{j}^{i}$ and $s_{j}^{i}=1-\tau d_{j}^{i}$. In contrast to the basic replicator Eq. (2), parameter $\tau$ cannot easily be removed from systems $S_{a}$ and $S_{b}$ by simple timescale adjustment. A similar situation occurs with the background payoff components $\Phi$ and $\Psi$, which simply cancel out in (2) but are still present in the population size equation. This will not be the case for systems $S_{a}$ and $S_{b}$. However, for simplicity, in this paper we do not deal explicitly with the background payoffs.

### Mixed PDE-ODE versions of systems $S_{a}$ and $S_{b}$

We can derive mixed PDE-ODE versions of systems $S_{a}$ and $S_{b}$, where the age profile is a continuous function, which are simpler and more mathematically elegant. The advantage is that they are independent of the timescale since the interaction rate $\tau$ will simply cancel out (see “Appendix E” for derivations). Thus the previous simplifying assumption about skipping it is obsolete in this case. Payoffs $d_{j}(t,l)$ and $f_{j}(t,l)$ are now continuous functions of the lifetime *l* and the strategic composition at time *t*. In addition the distinction between aggregated age class survival and game round survival discussed below Eq. (13) is not necessary since PDE versions of both systems will be driven by game payoffs only. Therefore for system $S_{a}$ we have

35 $$\begin{aligned} \frac{\partial a_{j}(t,l)}{\partial t}+\frac{\partial a_{j}(t,l)}{\partial l}= & {} a_{j}(t,l)\left[ -d_{j}(t,l)-(\bar{f}_{j}(t)\left( 1-\frac{n(t)}{K}\right) - \bar{d}_{j}(t))\right] , \end{aligned}$$

36 $$\begin{aligned} \dot{p}_{j}(t)= & {} p_{j}(t)\left( \left( \bar{f}_{j}(t)-\bar{f}(t)\right) \left( 1-\frac{n(t)}{K}\right) -\left( \bar{d}_{j}(t)-\bar{d}(t)\right) \right) , \end{aligned}$$

37 $$\begin{aligned} \dot{n}(t)= & {} n(t)(\bar{f}(t)\left( 1-\frac{n(t)}{K}\right) -\bar{d}(t)), \end{aligned}$$

with $$a_{j}(t,0)=\left( 1-\frac{n(t)}{K}\right) \bar{f}_{j}(t)$$, $$\bar{f} _{j}(t)=\int _{0}^{\infty }a_{j}(t,l)f_{j}(t,l)dl$$, $$\bar{d} _{j}(t)=\int _{0}^{\infty }a_{j}(t,l)d_{j}(t,l)dl$$, $$\bar{f}(t)=\sum _{j}$$ $$ p_{j}(t)\bar{f}_{j}(t)$$ and $$\bar{d}(t)=\sum _{j}p_{j}(t)\bar{d}_{j}(t)$$.

For system $S_{b}$ we have

38 $$\begin{aligned} \frac{\partial p_{j}(t,l)}{\partial t}+\frac{\partial p_{j}(t,l)}{\partial l}= & {} p_{j}(t,l)\left[ \bar{d}(t,l)-d_{j}(t,l)\right] , \end{aligned}$$

39 $$\begin{aligned} \frac{\partial a(t,l)}{\partial t}+\frac{\partial a(t,l)}{\partial l}= & {} a(t,l)\left[ -\bar{d}(t,l)-(\bar{f}(t)\left( 1-\frac{n(t)}{K}\right) -\bar{d}(t))\right] , \end{aligned}$$

40 $$\begin{aligned} \dot{n}(t)= & {} n(t)(\bar{f}(t)\left( 1-\frac{n(t)}{K}\right) -\bar{d}(t)), \end{aligned}$$

with $a(t,0)=\left( 1-\frac{n(t)}{K}\right) \bar{f}(t)$, $p_{j}(t,0)=\left( 1- \frac{n(t)}{K}\right) \int _{0}^{\infty }p_{j}(t,l)f_{j}(t,l)dl$, $\bar{d} (t,l)=\sum _{j}p_{j}(t)\bar{d}_{j}(t,l)$, $\bar{f}(t)=\int _{0}^{\infty }a(t,l) \bar{f}(t,l)dl$ and $\bar{d}(t)=\int _{0}^{\infty }a(t,l)\bar{d}(t,l)dl$, where $\bar{f}(t,l)=\sum _{j}p_{j}(t,l)\bar{f}_{j}(t,l)$ and $\bar{d}(t,l)=\sum _{j}p_{j}(t,l)\bar{d}_{j}(t,l)$.

### A sex ratio example

Now we will show how the methods presented in the previous sections can be used to extend the simpler age independent model to the age dependent case and how they can be used to model a sexually reproducing population. We will show this methodology by example of the synthetic sex ratio model (Argasinski 2012, 2013, 2017) combining simple explicit genetics (similar to the more advanced approaches as in Karlin and Lessard 1986) with rigorous strategic analysis. We will use the formulation of the model focused on selection of genes encoding sex ratio strategies (Argasinski 2013). Below we outline the basic details of this model. The introduction of the life cycle perspective to theoretical studies on the sex ratio is important, since data show the huge impact age specific mortalities can have on the dynamics of age specific sex ratios (for example see Orzack et al. 2015 for data showing the changes of the human sex ratio from conception to death).

We have a population consisting of *x* females and *y* males. All of them are carriers of a single gene encoding one from a finite number *w* of competing sex ratio strategies which are expressed by females (strategy $P_{j}\in [0,1]$ is carried by $x_{j}$ females and $y_{j}$ males and describes the fraction of male newborns in the brood of a female). Then the population state can be expressed by the population’s sex ratio $P=y/(x+y)$, primary sex ratio (average strategy of females) $\bar{P}_{pr}=\sum _{j=1}^{w} \dfrac{x_{j}}{x}P_{j}$ and vectors *G* and *M* where:

$G_{j}=\dfrac{x_{j}+y_{j}}{\sum _{z=1}^{w}\left( x_{z}+y_{z}\right) }$ the gene frequencies,

$M_{j}=\dfrac{y_{j}}{x_{j}+y_{j}}$ the sex ratios in the carrier subpopulations.

Then $P=\sum _{j=1}^{w}G_{j}M_{j}$ and $\bar{P}_{pr}=\sum _{j=1}^{w}\dfrac{ x_{j}}{x}P_{j}=\sum _{j=1}^{w}\dfrac{G_{j}(1-M_{j})}{1-P}P_{j}$. Strategy genes are inherited from mother or father with probability 0.5. The sex specific payoff functions describe the impact of direct reproductive success (offspring of the focal female or offspring of partners of the focal male) and the per capita normlized contribution of the same strategy carriers of the opposite sex. Therefore, the payoffs of male and female carriers and the average gene carrier are:

41 $$\begin{aligned} f_{m}(P_{j},G,M)= & {} \dfrac{k}{2}\left( \dfrac{x}{y}\bar{P}_{pr}+\dfrac{x_{j} }{y_{j}}P_{j}\right) \end{aligned}$$

42 $$\begin{aligned}= & {} \dfrac{k}{2}\left( \dfrac{1-P}{P}\bar{P}_{pr}+\dfrac{(1-M_{j})}{M_{j}} P_{j}\right) , \end{aligned}$$

43 $$\begin{aligned} f_{f}(P_{j},G,M)= & {} \dfrac{k}{2}\left( \left( 1-P_{j}\right) +\dfrac{y_{j}}{ x_{j}}\left( 1-\bar{P}_{pr}\right) \dfrac{x}{y}\right) \end{aligned}$$

44 $$\begin{aligned}= & {} \dfrac{k}{2}\left( \left( 1-P_{j}\right) +\dfrac{M_{j}}{(1-M_{j})}\left( 1-\bar{P}_{pr}\right) \dfrac{1-P}{P}\right) , \end{aligned}$$

45 $$\begin{aligned} f_{g}(P_{j},G,M)= & {} M_{j}f_{m}(P_{j},G,M)+(1-M_{j})f_{f}(P_{j},G,M) \end{aligned}$$

46 $$\begin{aligned}= & {} \dfrac{k}{2}\left[ M_{j}\dfrac{1-P}{P}+(1-M_{j})\right] \end{aligned}$$

where *k* is the number of offspring per female. The average payoffs are:

47 $$\begin{aligned} \bar{f}_{m}(G,M)= & {} k\dfrac{1-P}{P}\bar{P}_{pr}, \end{aligned}$$

48 $$\begin{aligned} \bar{f}(G,M)= & {} k\left( 1-P\right) . \end{aligned}$$

We can obtain the system describing the dynamics of gene frequencies and the sex ratios in the carrier subpopulations:

49 $$\begin{aligned} \dot{G}_{j}= & {} G_{j}\left( f_{g}(P_{j},G,M)-\bar{f}(G,M)\right) , \end{aligned}$$

50 $$\begin{aligned} \dot{M}_{j}= & {} M_{j}(f_{m}(P_{j},G,M)-f_{g}(P_{j},G,M)), \end{aligned}$$

leading to the following system of equations

51 $$\begin{aligned} \dot{G}_{j}= & {} G_{j}\left( \dfrac{1}{2}-P\right) \left( \dfrac{M_{j}}{P} -1\right) , \end{aligned}$$

52 $$\begin{aligned} \dot{M}_{j}= & {} \dfrac{k}{2}\left( M_{j}\left( \dfrac{1-P}{P}\right) \left( \bar{P}_{pr}-M_{j}\right) +\left( 1-M_{j}\right) \left( P_{j}-M_{j}\right) \right) . \end{aligned}$$

The above system can be regarded as an example of multi-level selection since the fate of a gene is determined by the actual composition of the carrier subpopulation described by the carriers’ sex ratio $M_{j}$ and the threshold between growth and decline is the adult sex ratio $P=\sum _{j=1}^{w}G_{j}M_{j}$. The parameters $M_{j}$ are determined by the Tug of War dynamics (52) describing the impact of female carriers producing newborns according to the carried strategy $P_{j}$ and randomly drawn female partners of male carriers producing newborns according to the average strategy of females $\bar{P}_{pr}$.

### The extension of the sex ratio model to the age structured case

**Fig. 3:**
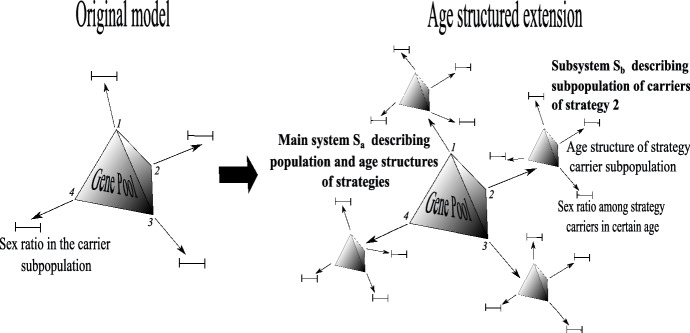
The extension of the phase space of the sex ratio model to the age structured case. The gene pool phase space is completed by respective subspaces describing the age structures among carriers of the particular genes, as in system $$S_{a}$$. Then each age structure subspace is completed by subspaces describing carriers’ sex ratios, according to system $$ S_{b}$$

We will extend this system in the following way (see Fig. 3).

System $$S_{a}$$ will be applied to extend the gene pool dynamics to the system with explicit age structure for each subpopulation of carriers (described by $$a_{j}^{i}$$ for the *j*-th gene) of the particular gene. This means that each Eq. (51) will be transformed to the form (21) and completed by the respective subsystem (20) describing the age structure of the subpopulation of carriers of the particular gene. In addition, for the age structure of each strategy we will apply system $$ S_{b}$$ to describe the dynamics of the sex ratios within each age class. Thus for each strategy, the respective subsystem (20) will be the core subsystem (28) of system $$S_{b}$$, and it will be completed by the respective subsystems (26,27), describing the dynamics of strategy carriers’ sex ratios in particular age classes. This structure will be the generalization of the $$M_{j}$$ Eq. (52) in the original model. Assume that survival, described by $$s_{f}^{i}$$ for females and by $$s_{m}^{i}$$ for males, depends only on sex and age. Males are active in the age classes from *a* to *b* and females from *c* to *d*, and fractions of sexually active female and male individuals carrying the *j* -th strategy are

53 $$\begin{aligned} S_{j}^{f}=\sum _{z=c}^{d}a_{j}^{z}\big (1-M_{j}^{z}\big ),\quad S_{j}^{m}=\sum _{z=a}^{b}a_{j}^{z}M_{j}^{z}{.} \end{aligned}$$

Analogous parameters for the whole population are

54 $$\begin{aligned} \bar{S}^{f}=\sum _{j=1}^{w}G_{j}S_{j}^{f},\bar{S} ^{m}=\sum _{j=1}^{w}G_{j}S_{j}^{m}. \end{aligned}$$

We also have $P=\sum _{j=1}^{w}G_{j}\sum _{i}a_{j}^{i}M_{j}^{i}$ , and the primary sex ratio is:

55 $$\begin{aligned} \bar{P}_{pr}=\sum _{j=1}^{w}\dfrac{G_{j}S_{j}^{f}}{\sum _{z}G_{z}S_{z}^{f}} P_{j}=\dfrac{\sum _{j=1}^{w}G_{j}S_{j}^{f}P_{j}}{\bar{S}^{f}}. \end{aligned}$$

Thus this is the average strategy of active females describing the proportion of males among all newborns or zygotes. The operational sex ratio among active carriers of strategy *j* and the equivalent average value for the population is

56 $$\begin{aligned} M_{j}^{op}=\dfrac{S_{j}^{m}}{S_{j}^{m}+S_{j}^{f}},P_{op}= \dfrac{\bar{S}^{m}}{\bar{S}^{m}+\bar{S}^{f}}. \end{aligned}$$

The equations on *G* should be updated according to the additional assumptions on age limits of sexual activity (age classes from *a* to *b* for males and *c* to *d* for females). We should also derive the respective forms of per capita fertility payoffs described in the new coordinates. For derivation of the dynamics we need the following operational male fertility payoff of active males, average per capita gene fertility payoff and the average fertility in the whole population (the detailed derivation is in “Appendix F”):

57 $$\begin{aligned} f_{m}^{op}(P_{j},a,G,M)= & {} \dfrac{k}{2}\left( \dfrac{1-P_{op}}{P_{op}}\bar{P} _{pr}+\dfrac{1-M_{j}^{op}}{M_{j}^{op}}P_{j}\right) , \end{aligned}$$

58 $$\begin{aligned} f_{g}(P_{j},a,G,M)= & {} \dfrac{k}{2}\left( S_{j}^{f}+S_{j}^{m}\dfrac{1-P_{op}}{ P_{op}}\right) , \end{aligned}$$

59 $$\begin{aligned} \bar{f}(a,G,M)= & {} k\bar{S}^{f}. \end{aligned}$$

Note that $\left( 1-P_{op}\right) /P_{op}$ describes the number of partners and $\left( 1-M_{j}^{op}\right) /M_{j}^{op}$ the number of female carriers (“sisters”) of the average male carrier of the focal strategy gene. Therefore, the male operational fertility payoff $f_{m}^{op}$ describes the fertility of their partners with the average strategy and “sisters” carrying the same gene. The gene payoff $f_{g}$ describes the aggregated fertility of all female carriers and all partners of male carriers. Thus we will obtain the following general system derived in “Appendix G”:

60 $$\begin{aligned} \dot{G}_{j}= & {} G_{j}\left( \left( f_{g}(P_{j},a,G,M)-\bar{f}(a,G,M)\right) \left( 1-\dfrac{n}{K}\right) +\left( \bar{s}_{j}-\bar{s}\right) \right) , \end{aligned}$$

61 $$\begin{aligned} \dot{a}_{j}^{i}= & {} a_{j}^{i-1}\bar{s}_{j}^{i-1}-a_{j}^{i}\left[ f_{g}(P_{j,}a,G,M)\left( 1-\dfrac{n}{K}\right) +\bar{s}_{j}\right] , \end{aligned}$$

62 $$\begin{aligned} \dot{M}_{j}^{0}= & {} \dfrac{\left( f_{m}^{op}(P_{j},a,G,M)S_{j}^{m}-M_{j}^{0}f_{g}(P_{j},a,G,M)\right) }{ a_{j}^{0}}\left( 1-\dfrac{n}{K}\right) , \end{aligned}$$

63 $$\begin{aligned} \dot{M}_{j}^{i}= & {} \dfrac{a_{j}^{i-1}}{a_{j}^{i}}\left( M_{j}^{i-1}s_{m}^{i-1}-M_{j}^{i}\bar{s}_{j}^{i-1}\right) , \end{aligned}$$

64 $$\begin{aligned} n= & {} n\left( \bar{f}\left( 1-\dfrac{n}{K}\right) +\bar{s}-1\right) , \end{aligned}$$

where $\bar{s}_{j}^{i}=M_{j}^{i}s_{m}^{i}+\left( 1-M_{j}^{i}\right) s_{f}^{i}$ describes the average survival of the carrier of the *j*th strategy determined by the actual carriers sex ratio. Then $\bar{s} _{j}=\sum _{i=0}^{m}a_{j}^{i}\bar{s}_{j}^{i}$ and $\bar{s} =\sum _{j=1}^{w}G_{j}\bar{s}_{j}$. Thus the general Eqs. (20–22) have become Eqs. (60, 61, 64) through the sequences: (21)$\rightarrow$ (49)$\rightarrow$ (60) , (20)$\rightarrow$ (28)$\rightarrow$ (61), (26)$\rightarrow$ (62), (27)$\rightarrow$ (63). Figure 3 shows how the phase space of the original model was extended to the age structured case. After substitution of the payoff functions (see “Appendix G”) we obtain the system:

65 $$\begin{aligned} \dot{G}_{j}= & {} G_{j}\left( k\left[ \dfrac{1}{2}\left( \dfrac{S_{j}^{f}}{\bar{ S}^{f}}+\dfrac{S_{j}^{m}}{\bar{S}^{m}}\right) -1\right] \bar{S}^{f}\left( 1- \dfrac{n}{K}\right) +\left( \bar{s}_{j}-\bar{s}\right) \right) , \end{aligned}$$

66 $$\begin{aligned} \dot{a}_{j}^{i}= & {} a_{j}^{i-1}\bar{s}_{j}^{i-1}-a_{j}^{i}\left[ \dfrac{k}{2} \left( S_{j}^{f}+S_{j}^{m}\dfrac{\bar{S}^{f}}{\bar{S}^{m}}\right) \left( 1- \dfrac{n}{K}\right) +\bar{s}_{j}\right] , \end{aligned}$$

67 $$\begin{aligned} \dot{M}_{j}^{0}= & {} \dfrac{k}{2a_{j}^{0}}\left( S_{j}^{m}\left( \bar{P} _{pr}-M_{j}^{0}\right) \dfrac{\bar{S}^{f}}{\bar{S}^{m}}+S_{j}^{f}\left( P_{j}-M_{j}^{0}\right) \right) \left( 1-\dfrac{n}{K}\right) , \end{aligned}$$

68 $$\begin{aligned} \dot{M}_{j}^{i}= & {} \dfrac{a_{j}^{i-1}}{a_{j}^{i}}\left( M_{j}^{i-1}s_{m}^{i-1}-M_{j}^{i}\bar{s}_{j}^{i-1}\right) , \end{aligned}$$

69 $$\begin{aligned} n= & {} n\left[ k\bar{S}^{f}\left( 1-\dfrac{n}{K}\right) +\bar{s}-1\right] , \end{aligned}$$

where average survival probabilities are

70 $$\begin{aligned}&\bar{s}_{j}^{i}=M_{j}^{i}s_{m}^{i}+\big (1-M_{j}^{i}\big )s_{f}^{i},\bar{ s}_{j}=\sum _{i=1}^{m}a^{i}\bar{s}_{j}^{i}. \nonumber \\&S_{j}^{f}=\sum _{z=c}^{d}a_{j}^{z}\big (1-M_{j}^{z}\big ),\text { }\bar{S} ^{f}=\sum _{j=1}^{w}G_{j}S_{j}^{f},\text { }S_{j}^{m}= \sum _{z=a}^{b}a_{j}^{z}M_{j}^{z},\text { }\bar{S}^{m}= \sum _{j=1}^{w}G_{j}S_{j}^{m}\nonumber \\ \end{aligned}$$

are the fractions of sexually active females and males among the $P_{j}$ gene carriers, and the respective averages. Thus the selection mechanism is seriously altered by the age structure. The above system shows that differences in mortality between sexes and different ages of sexual activity can significantly affect the selection of individual strategies. Equation (67) contain the terms $S_{j}^{m}$ and $S_{j}^{f}$ describing the fractions of sexually active individuals and are the equivalent of the Tug of War dynamics (52). The dynamics of the age structure of each strategy is attracted by

71 $$\begin{aligned} \hat{a}_{j}^{i}=\hat{a}_{j}^{i-1}\frac{M_{j}^{i-1}s_{m}^{i-1}+\left( 1-M_{j}^{i-1}\right) s_{f}^{i-1}}{\dfrac{k}{2}\left( S_{j}^{f}+S_{j}^{m} \dfrac{\bar{S}^{f}}{\bar{S}^{m}}\right) \left( 1-\dfrac{n}{K}\right) +\bar{s} _{j}}. \end{aligned}$$

Sex ratios among the *j*th strategy carriers of particular ages converge to

72 $$\begin{aligned} \hat{M}_{j}^{0}=\frac{\bar{P}_{pr}\dfrac{S_{j}^{m}}{\bar{S}^{m}}\bar{S} ^{f}+S_{j}^{f}P_{j}}{\dfrac{S_{j}^{m}}{\bar{S}^{m}}\bar{S}^{f}+S_{j}^{f}}, \hat{M}_{j}^{i}=\frac{M_{j}^{i-1}s_{m}^{i-1}}{\bar{s}_{j}^{i-1} }i>0. \end{aligned}$$

Note that when we assume that there are no differences in survival probabilities between sexes ($$s_{f}^{i}=s_{m}^{i}$$) then the system (65–69) reduces to the simplified version. Equation (66) will be independent of parameters $$M_{j}^{i}$$ and the Eq. (68) will converge to a constant value over the whole life cycle ($$ M_{j}^{i}=M_{j}^{0}$$ for all *i*). Therefore all strategies will have the same age structure. In effect the bracketed term $$\left( \bar{s}_{j}-\bar{s} \right) $$, describing the excess of the mortality payoff from average mortality, will vanish in Eq. (65) and selection of the genes will be driven by the excess fertility payoff bracket $$\left( f_{g}(P_{j},a,G,M)-\bar{f}(a,G,M)\right) $$ describing the Fisherian mechanism driven by the difference in reproductive value between the sexes;

73 $$\begin{aligned} \left( f_{g}(P_{j},a,G,M)-\bar{f}(a,G,M)\right) =k\left[ \dfrac{1}{2}\left( \dfrac{S_{j}^{f}}{\bar{S}^{f}}+\dfrac{S_{j}^{m}}{\bar{S}^{m}}\right) -1 \right] \bar{S}^{f}, \end{aligned}$$

which is equivalent to the Shaw–Mohler formula (Shaw and Mohler 1953). If we assume that both sexes are mature in the same age classes then $$ S_{j}^{f}=1-S_{j}^{m}$$, we have that operational sex ratios (56) are $$M_{j}^{op}=S_{j}^{m}$$and $$P_{op}=\bar{S}^{m}$$ ($$\bar{S} ^{f}=1-P_{op}$$). Therefore for the operational sex ratio $$P_{op}=0.5$$ the above formula equals zero for all strategies. When we additionally assume that the sex specific survivals for different ages are the same, the system replicates the results of the original model . In the general case if $$ S_{j}^{f}=\bar{S}^{f}$$ and $$S_{j}^{m}=\bar{S}^{m}$$ then obviously the operational sex ratios (56) are equal. In other cases, the strategies with the greater fraction of the sex which is in the minority among active individuals (according to the operational sex ratio $$P_{op}$$) will have a greater value of (73). Since all individuals of the same sex suffer the same mortality, the values of parameters $$ S_{j}^{f}$$ and $$S_{j}^{m}$$ are determined by the allocation of sexes at birth, determined by their encoded strategy. Due to the constant brood size *k*, an increase of female newborns leads to a decrease of male newborns and vice versa. Therefore, this allocation will determine operational sex ratios and selection should act accordingly to differences in operational sex ratios, similarly to (51).

**Fig. 4:**
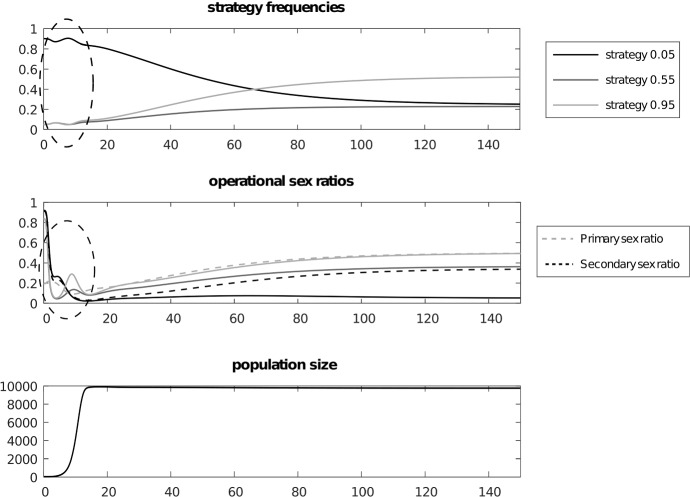
Panel **a** dynamics of gene frequencies, panel **b** operational sex ratios, for strategies $$M_{0.05}^{op}$$, $$M_{0.55}^{op}$$, $$M_{0.95}^{op}$$ and primary and operational sex ratios of the population $$\bar{P}_{pr}$$ and $$ P_{op}$$, panel **c** population size. Trajectories show that $$P_{op}$$ is the threshold between growth and decline of the gene frequency depending on the value of $$M_{j}^{op}$$. This is shown by the example of strategy 0.05, where bumps in the marked areas are caused by two types of events. The first is when the strategy’s operational sex ratio $$M_{j}^{op}$$ passes the population’s operational sex ratio $$P_{op}$$, which is the threshold between growth and decline. The second is when the average operational sex ratio $$ P_{op}$$ passes the value of 0.5 which inverts the strategic situation, since the opposite sex is in the minority when this happens

**Fig. 5:**
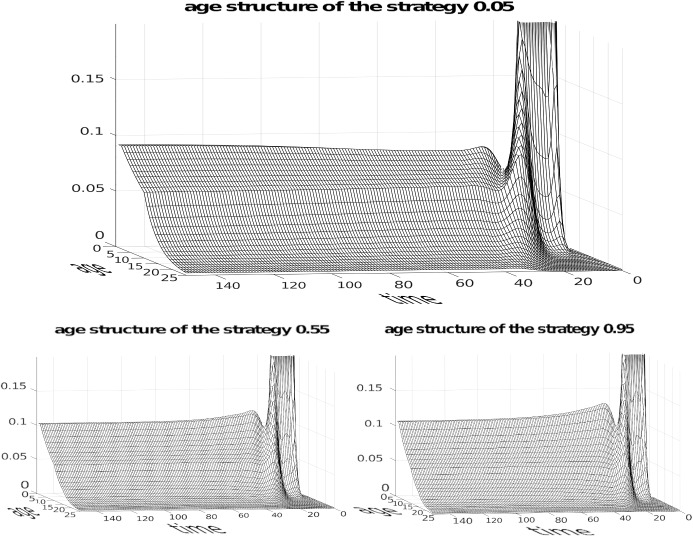
Trajectories of age classes. The initial behaviour is caused by huge differences in the initial sex ratios. The assumed changes in age specific survivals slightly affect the trajectories

**Fig. 6:**
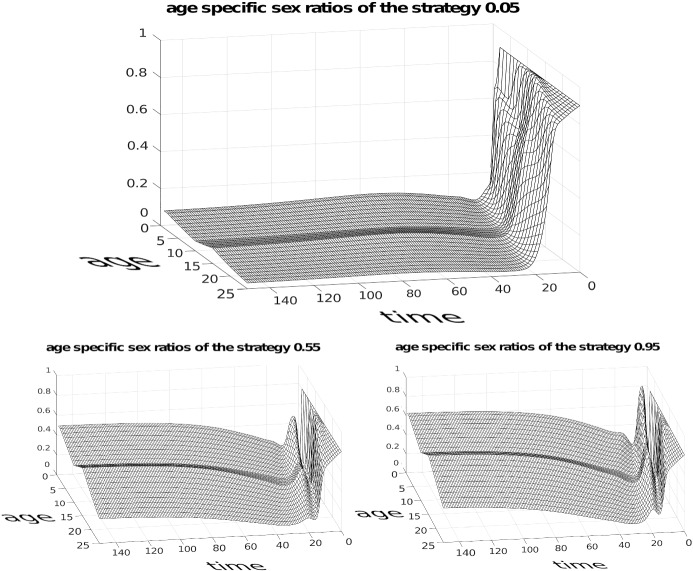
Trajectories of age specific sex ratios. The pattern caused by the assumed changes in survival probabilities is clearly visible

We can see this in Fig. 4 depicting a numerical simulation for the case of three competing strategies $$P_{1}=0.05$$, $$P_{2}=0.55$$, $$P_{3}=0.95$$ with 25 age classes plus infant age class 0. For simplicity we assumed that age class survivals will be the same with only one change at some arbitrary age, different for males and females. For females we have survival 0.95 until age 10 and 0.80 in subsequent ages. For males we have 0.88 until age 15 and then 0.72 subsequently. By definition survival in the last age classes is zero. Females are fertile from age $$c=8$$ until age $$d=15$$ while males are active from age $$a=8$$ until age $$b=20$$. The initial population size was $$n(0)=40$$ with a carrying capacity *K* = 10 000. Initial conditions are $$G_{1}(0)=0.9$$, $$G_{2}(0)=G_{3}(0)=0.05$$, $$M_{1}^{0}=0.7$$ and $$ M_{2}^{0}=M_{3}^{0}=0.1$$. We start from a very young population where adult age classes have frequencies 0.001 leading to a 0.025 proportion of non-infant individuals and sex ratios are $$M_{1}^{i}=0.9$$ and $$ M_{2}^{i}=M_{3}^{i}=0.8$$. These exaggerated conditions show the initial dynamics of the growing cohort leading to the interesting patterns depicted in Fig. 5 depicting the age structure and Fig 6 showing the dynamics of age specific sex ratios.

**Fig. 7:**
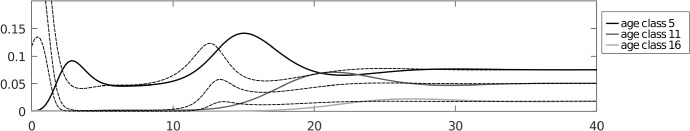
A plot of the convergence to the respective Euler–Lotka manifolds (dashed lines) for arbitrarily chosen age classes for strategy 0.05. The convergence is delayed by some inertia caused by the age dynamics

**Fig. 8:**
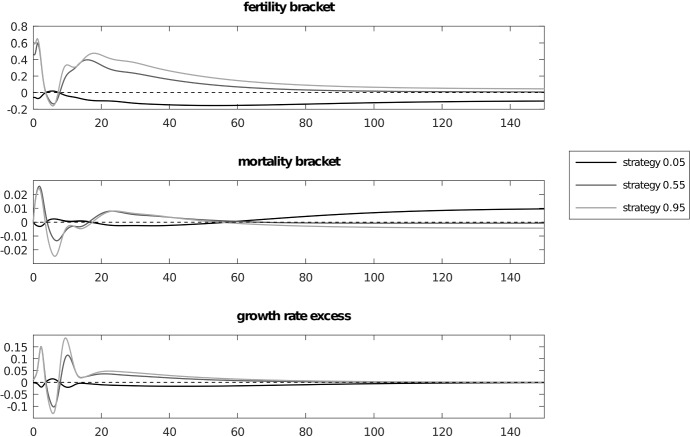
Plots of the excess fertility payoffs $\left( f_{g}(P_{j},a,G,M)-\bar{f}(a,G,M)\right)$, excess mortality payoffs $\left( \bar{s}_{j}-\bar{s}\right)$ and the gene frequency growth rates $\left( f_{g}-\bar{f}\right) \left( 1-n/K\right) +\left( \bar{s}_{j}-\bar{s} \right)$ from the gene pool dynamics (65). Fertility payoffs are not equal as in the classical theory, and the same situation is true for mortality payoffs, but the right hand sides of the equations are zero. This shows that the explanation for the primary sex ratio being 0.5 needs an explicit consideration of the interplay between fertility and mortality

Figure 7 shows the delayed convergence to the respective Euler–Lotka manifolds. At the global equilibrium excess fertility payoffs (73) (the difference between the payoff and the mean) are not equal to zero because they must balance the nonzero values of the excess survival payoffs for growth rates to be equal (Fig. 8). Therefore, the classical Fisherian equilibrium focused only on fertility payoffs is not reached here. A question arises about the interplay between fertility and survival and how it leads to the primary sex ratio of 0.5. In addition the operational sex ratio is far from 0.5. Therefore in this case the Fisherian mechanism is not enough to explain the origins of the primary sex ratio of 0.5. Figure 4 shows that the mechanism driven by the operational sex ratios still works but all values are rescaled, and we also have different mortalities for different strategies. The interplay between the Fisherian mechanism, driven by fertility and differences in reproductive value between the sexes, and age structure, driven by survival differences between the sexes, needs an explanation which will be the subject of future work.

## Discussion

In this work we presented a new modelling framework combining evolutionary dynamics with demographic structure. This approach can be a useful tool in the development of the synthesis between evolutionary game theory and life history theory. We started with the derivation of the ODE discretized approximation of the McKendrick von Foerster model of age structured populations and its critical manifold equivalent to the Euler–Lotka equation. This was extended to the explicit case of multiple competing strategies and transformed into two types of age structured replicator dynamics. The first focused on the selection of strategies when each strategy is described by a subsystem describing the dynamics of the age structure. The second focused on the age structure of the whole population and a subsystem of the strategies within each age class. These led to huge ODE systems which are equivalent to systems of Bernadelli–Lewis–Leslie matrices. Another complication is that for the discretized age structure we need age class survival functions which will describe the aggregated outcomes of all interactions (game rounds) that have happened during a single time unit. This survival function is distinct from game round survival which can be used for the derivation of the fertility-survival trade-off functions used in situations when only survivors of the interaction can reproduce (Argasinski and Broom 2012, 2018a, b). In addition we have outlined the PDE versions of the obtained systems to indicate a future direction of research.

Both approaches are combined in the illustrative example of a sex ratio model. This is an extension of the dynamic sex ratio model (Argasinski 2012, 2013, 2017). It shows that when we assume different mortalities for both sexes, the classical Fisherian explanation based on the differences of reproductive values of offspring is not enough to explain convergence to the primary sex ratio of 0.5. The excess fertility payoff does not converge to 0 which would be equivalent to an equal reproductive values for both sexes, but its non-zero value is equal to the value of the excess survival payoff. The question of how this mechanism works in detail should be explained in future research. The new model provides a theoretical framework that can be used to explain the mechanisms shaping the patterns observable in data collected on age specific sex ratios from conception to death, as in Orzack et al. (2015) and Orzack (2016).

The obtained results clearly show that a life cycle perspective plays a crucial role in evolutionary processes. In the classical approaches to evolutionary game theory individuals cannot change their properties during their lifetime. Thus their life history is a memoryless process, and survival of a single interaction does not change the state of the individual. This is caused by the fact that the classical approaches to evolutionary games are focused on the strategies interpreted as patterns of behaviour, not on the individual itself. The exception to this rule is the state based approach (Houston and McNamara 1999; Argasinski and Rudnicki 2020). The explicit description of the life cycle and the different payoffs at different ages leads to a more complicated game theoretic structure. In particular a mixed PDE-ODE approach will lead to more complex payoff functions based on continuous distributions of ages for different strategies. This will need more sophisticated methods, such as models with function valued traits (Oechssler and Riedel 2001; Dieckmann et al. 2006, van Veelen and Spreij 2009), state based games (Houston and McNamara 1991, 1999; Argasinski and Rudnicki 2020) or “large games” with a distinction between strategy sets and population states (Wieczorek and Wiszniewska 1998; Wieczorek 2004, 2005), as opposed to basic two person matrix games. The modelling framework proposed in this paper can also be a useful tool in the research on animal personalities The combination of game theoretic analysis with an explicit age structure will allow us to analyze the relationships between behavioural strategies (such as aggression or cowardice) and life history traits (such as allocation of energy into growth or reproduction). This is important because life history trade-offs are shaped by external mortality which is the outcome of interactions with the environment. On the other hand, the demographic outcomes of interactions such as mortality are affected by phenotypic traits such as growth shaped by life history strategies (Wolf and Weissing 2010; Wolf and McNamara 2012). This constitutes life-history-behavioural feedback.

## Appendix A: Derivation of the frequency equations

(1) *Discretization of the of McKendrick von Foerster model* (10) We need to divide the continuous time into separate discrete unit compartments describing age classes consisting of individuals of ages from the interval $$(l,l+1]$$. Recall that game rounds occur at intensity $$\tau $$. Note that the exponential dynamics emerges from an aggregation of survival from some independent interactions, when the focal individual survives (or not) several events and the aggregated survival is the product of survival probabilities of those events. However we can imagine a timescale where interactions are sufficiently rare, so that only a small fraction of individuals play a single round of the game. Then the dynamics is technically linear and survival is described by the first term of the Taylor series of the exponential function. Thus assume that time interval $$dt=dl=1$$ is small enough that a small fraction $$\tau d(t,l)dt$$ of individuals will die due to aggregated outcomes of independent game rounds. The remaining $$1-$$$$\tau d(t,l)dt$$ survivors will be moved to the next age compartment. Then from Eq. (9) we have that $$n(t+1,l+1)=\left( 1-\tau d(t,l)\right) n(t,l)=s(t,l)n(t,l)$$ which describes the move from point *t*, *l* to point $$t+1,l+1$$. Assume that during a unit interval all surviving individuals from age *l* will be moved to age $$l+1$$ while all individuals from $$l+1$$ will be moved from this age to the next age step or die. Therefore during a single time unit we have linear movement occurring at incoming rate $$s(t,l-1)n(t,l-1)$$ and removal rate -*n*(*t*, *l*), since all individuals will be removed during a single time unit. Therefore this linear process can be well approximated by the first order Taylor expansion for $$ \Delta t=1$$ where the bracketed term can be interpreted as the first derivative;

74 $$\begin{aligned} n(t+1,l)=n(t,l)+[s(t,l-1)n(t,l-1)-n(t,l)], \end{aligned}$$

therefore the bracketed term constitutes derivative *dn*/*dt*, leading to

$$\begin{aligned} \frac{dn(t,l)}{dt}=s(t,l-1)n(t,l-1)-n(t,l). \end{aligned}$$

Note that (74) can be presented in the form $$ n(t+1,l)=s(t,l-1)n(t,l-1)$$ which leads to the Leslie matrix (7). When we change notation to the numbered age classes describing age increments and assume that aggregated survival rate $$s^{i}(t)$$ and fertility rate $$\tau f^{i}\left( t\right) $$ can change in time, we obtain the system

75 $$\begin{aligned}&\dot{n}^{0}(t)=\sum _{i=0}^{m}n^{i}(t)\tau f^{i}(t)-n^{0}(t), \end{aligned}$$

76 $$\begin{aligned}&\dot{n}^{i}(t)=s^{i-1}(t)n^{i-1}(t)-n^{i}(t)i=1,\ldots ,m. \end{aligned}$$

It is reasonable to assume that $$s^{0}=1$$, (other values are equivalent to $$ s^{0}=1$$ with rescaled fertilities $$f^{i}$$) and $$s^{m}=0$$.

However, to be compatible with the replicator dynamics and game theoretic machinery, the dynamics should be expressed in terms of phenotype frequencies. Let us change the coordinates to the frequencies $a^{i}=n^{i}/n$ (with $n=\sum _{i=0}^{m}n^{i}$) describing the age structure. The system (,76) can be presented in the form of the Malthusian equations:

77 $$\begin{aligned} \dot{n}^{0}(t)= & {} \sum _{i=0}^{m}n^{i}(t)\tau f^{i}(t)-n^{0}(t)=n^{0}(t)\left( \sum _{i=0}^{m}\dfrac{n^{i}(t)\tau f^{i}(t)}{ n^{0}(t)}-1\right) \nonumber \\= & {} n^{0}(t)\left( \sum _{i=0}^{m}\dfrac{a^{i}(t)\tau f^{i}(t)}{a^{0}(t)} -1\right) , \end{aligned}$$

78 $$\begin{aligned} \dot{n}^{i}(t)= & {} s^{i-1}(t)n^{i-1}(t)-n^{i}(t)=n^{i}(t)\left( \dfrac{ n^{i-1}(t)s^{i-1}(t)}{n^{i}(t)}-1\right) \nonumber \\= & {} n^{i}(t)\left( \dfrac{a^{i-1}(t)}{a^{i}(t)}s^{i-1}(t)-1\right) i=1,\ldots ,m. \end{aligned}$$

Therefore, this system can be presented as a system of frequency dependent replicator equations $\dot{a}^{i}=a^{i}(r^{i}-\bar{r})$ and a single equation on the scaling parameter $\dot{n}=n\bar{r}$. Since $\sum _{i=0}^{m}a^{i}=1$ and $s^{m}=0$ we have the average Malthusian growth rate as

79 $$\begin{aligned} \bar{r}=a^{0}\left( \sum _{i=0}^{m}\dfrac{a^{i}\tau f^{i}}{a^{0}}-1\right) +\sum _{i=1}^{m}a^{i}\left( \dfrac{a^{i-1}s^{i-1}}{a^{i}}-1\right) =\sum _{i=0}^{m}a^{i}\left( \tau f^{i}+s^{i}\right) -1.\nonumber \\ \end{aligned}$$

Then we can formulate a system of frequency dependent replicator equations by transforming Eq. (78) for $i=1,\ldots ,m$ (the equation for 0 is redundant and can be removed and $a^{0}=1-\sum \limits _{i=1}^{m}a^{i}$):

80 $$\begin{aligned} \dot{a}^{i}= & {} a^{i}\left( \dfrac{a^{i-1}}{a^{i}}s^{i-1}-1-\bar{r}\right) =a^{i-1}s^{i-1}-a^{i}\left( \sum _{k=0}^{m}a^{k}\left( \tau f^{k}+s^{k}\right) \right) \end{aligned}$$

81 $$\begin{aligned} \dot{n}= & {} n\bar{r}=n\left( \sum _{i=0}^{m}a^{i}\left( \tau f^{i}+s^{i}\right) -1\right) . \end{aligned}$$

To add density dependence we should multiply the fertility rate by the logistic suppression coefficient $\left( 1-\dfrac{n}{K}\right)$.

(2) *Frequency and density dependence and the choice of time unit determining the discretization step* When the above system describes the dynamics of the age structure of the subpopulation of carriers of some strategy competing with other strategies (indexed by subscripts) then the parameters $s_{j}^{i}(t)=1-\tau d_{j}^{i}(p(t),a(t))$ and $\tau f_{j}^{i}\left( p(t),a(t)\right)$ (thus $r_{j}=\tau \tilde{r} _{j}=\tau \sum _{i=0}^{m}a_{j}^{i}\left( f_{j}^{i}(1-n/k)-d_{j}^{i}\right)$) are game payoffs depending on strategy frequencies $p_{j}=n_{j}/ \sum _{k}n_{k}$ (and their age distributions, but now we limit our reasoning to strategy frequencies *p*). We want to choose the longest possible time step to reduce the number of equations. However the frequencies will change in time, so the discretization step cannot be too big, since the aggregated payoffs during unit interval will depend on the changes of $\tau d_{j}^{i}(p(t))$ and $\tau f_{j}^{i}(p(t))$ during that time interval. The time unit should be short enough that these vital rates will not change significantly and the number of individuals will change nearly linearly within each age class. Thus we should analyze how much the strategy frequencies $p_{j}$ can change during unit time and how this affects the vital rates. For small $\Delta t=1$ we have a change of

$$\begin{aligned} \Delta n_{j}= & {} n_{j}(t)\tau \tilde{r}_{j}(t)\Delta t =\sum _{i}n_{j}^{i}(t)\tau \left( f_{j}^{i}(p(t))\left( 1-\dfrac{n}{K}\right) -d_{j}^{i}(p(t))\right) \Delta t\ \end{aligned}$$

(positive or negative) for each *j*, leading to the change $\Delta n=\sum _{j}\Delta n_{j}=n(t)\tau \tilde{r}(t)\Delta t$ for the population size. Then

$$\begin{aligned} p_{j}(t+1)= & {} \frac{n_{j}(t)}{n(t)+\Delta n}+\frac{\Delta n_{j}}{n(t)+\Delta n}=\frac{n_{j}(t)}{n(t)}\frac{n(t)}{n(t)+\Delta n}+\frac{\Delta n_{j}}{ n(t)+\Delta n} \\= & {} \frac{n_{j}(t)}{n(t)}\left[ 1-\frac{\Delta n}{n(t)+\Delta n}\right] + \frac{\Delta n_{j}}{n(t)+\Delta n}=p_{j}(t)+\frac{\Delta n_{j}-p_{j}(t)\Delta n}{n(t)+\Delta n}. \end{aligned}$$

Therefore $p_{j}(t+1)=p_{j}(t)+\Delta p_{j}$ where vector $\Delta p$ consists of

82 $$\begin{aligned} \Delta p_{j}(\tau )=\dfrac{\Delta n_{j}-p_{j}(t)\Delta n}{n(t)+\Delta n}= \dfrac{\tau }{1+\tau \tilde{r}(t))}p_{j}(t)\left( \tilde{r}_{j}(t)-\tilde{r} (t)\right) . \end{aligned}$$

Thus the pace of increment is nearly linear or slower since $\tau /(1+\tau \tilde{r}(t))<\tau$. We can set the timescale by adjusting parameter $\tau$ in the formulae $\Delta f_{j}^{i}=f_{j}^{i}(p(t)+\Delta p(\tau ))-f_{j}^{i}(p(t))$ and $\Delta d_{j}^{i}=d_{j}^{i}(p(t)+\Delta p(\tau ))-d_{j}^{i}(p(t))$ to make their values small enough that $\tau \Delta f_{j}^{i}$ and $\tau \Delta d_{j}^{i}$ are negligible. For the density factor we have that $\left( 1-n(t+1)/K\right) =\left( 1-(n(t)+\Delta n)/K\right)$ and the change of fertility rate is $-\tau f_{j}^{i}(p(t))\Delta n/K$, thus it depends on $\tau /K$ and is negligible for even big time steps.

## Appendix B: The stationary age distribution and the Euler–Lotka equation in the continuous case

From (80) (recall that $\tau$ is hidden in the fertilities $f^{i}$), for the non-infant age classes ($i>0$) the stationary points for the age structure of this system are:

83 $$\begin{aligned} \dfrac{\hat{a}^{i-1}s^{i-1}}{\hat{a}^{i}}=\sum _{i=0}^{m}\hat{a}^{i}\left( f^{i}+s^{i}\right) i=1,\ldots ,m, \end{aligned}$$

therefore

84 $$\begin{aligned}&\hat{a}^{i}=\dfrac{\hat{a}^{i-1}s^{i-1}}{\sum _{i=0}^{m}\hat{a}^{i}\left( f^{i}+s^{i}\right) }=\dfrac{\hat{a}^{i-1}s^{i-1}}{\bar{r}(\hat{a})+1} \Rightarrow \end{aligned}$$

85 $$\begin{aligned}&\hat{a}^{i}=\dfrac{\hat{a}^{0}\prod \limits _{z=0}^{i-1}s^{z}}{\left( \bar{r}( \hat{a})+1\right) ^{i}}. \end{aligned}$$

Then $\sum _{i=0}^{m}\hat{a}^{i}=1$ implies that

86 $$\begin{aligned} \hat{a}^{0}\left( \sum _{i=0}^{m}\dfrac{\prod \limits _{z=0}^{i-1}s^{z}}{\left( \bar{r}(\hat{a})+1\right) ^{i}}\right) =1\Rightarrow \hat{a}^{0}=\left( \sum _{i=0}^{m}\dfrac{\prod \limits _{z=0}^{i-1}s^{z}}{\left( \bar{r}(\hat{a} )+1\right) ^{i}}\right) ^{-1}. \end{aligned}$$

(note the similarity to the Euler–Lotka equation). The stable age structure is a unique vector of frequencies among age classes, conditional on the average Malthusian growth rate of the population. Now let us prove the equivalence with the Euler–Lotka equation. After substitution of the stable age frequencies from Eq. (85) into Eq. (77) we obtain:

87 $$\begin{aligned} \dot{n}^{0}=n^{0}\left( \sum _{i=0}^{m}\dfrac{\hat{a}^{i}f^{i}}{\hat{a}^{0}} -1\right) =n^{0}\left( \sum _{i=0}^{m}\dfrac{f^{i}\prod \limits _{z=0}^{i-1}s^{z}}{\left( \bar{r}(\hat{a})+1\right) ^{i}}-1\right) . \end{aligned}$$

Frequency equilibrium implies that per capita growth rates in all age classes are equal to the average growth rate $\bar{r}(\hat{a})$. Thus equality will also be satisfied for the growth rate of the 0 age class, leading to

88 $$\begin{aligned} \sum _{i=0}^{m}\dfrac{f^{i}\prod \limits _{z=0}^{i-1}s^{z}}{\left( \bar{r}(\hat{ a})+1\right) ^{i}}-1=\bar{r}(\hat{a})\Rightarrow \sum _{i=0}^{m}\dfrac{ f^{i}\prod \limits _{z=0}^{i-1}s^{z}}{\left( \bar{r}(\hat{a})+1\right) ^{i+1}} =1, \end{aligned}$$

which is the Euler–Lotka equation.

## Appendix C: Derivation of system $S_{a}$

We start from the Malthusian system describing the dynamics of age classes in the subpopulation of carriers of the *j*-th strategy:

89 $$\begin{aligned} \dot{n}_{j}^{0}= & {} \sum _{i=0}^{m}n_{j}^{i}f_{j}^{i}-n_{j}^{0}, \end{aligned}$$

90 $$\begin{aligned} \dot{n}_{j}^{i}= & {} s_{j}^{i-1}n_{j}^{i-1}-n_{j}^{i}. \end{aligned}$$

According to (11) the above system can be transformed into the frequency replicator dynamics of age classes:

91 $$\begin{aligned} \dot{a}_{j}^{i}=a_{j}^{i-1}s_{j}^{i-1}-a_{j}^{i}\left( \sum _{k=0}^{m}a_{j}^{k}\left( f_{j}^{k}+s_{j}^{k}\right) \right) . \end{aligned}$$

The Malthusian equation describing the growth of *j*-strategists is

92 $$\begin{aligned} \dot{n}_{j}=n_{j}\bar{r}_{j}(a_{j})=n_{j}\left( \sum _{i=0}^{m}a_{j}^{i}\left( f_{j}^{i}+s_{j}^{i}\right) -1\right) . \end{aligned}$$

Then the replicator dynamics for the changes of strategy frequencies are

93 $$\begin{aligned} \dot{p}_{j}=p_{j}\left( \bar{r}_{j}-\bar{r}\right) =p_{j}\left( \left( \bar{f }_{j}-\bar{f}\right) +(\bar{s}_{j}-\bar{s})\right) , \end{aligned}$$

where $\bar{r}=\sum _{j=1}^{w}p_{j}\bar{r}_{j}$ , $\bar{f}_{j}= \sum _{i=0}^{m}a_{j}^{i}f_{j}^{i}$, $\bar{f}=\sum _{j=1}^{w}p_{j}\bar{f}_{j}$, $\bar{s}_{j}=\sum _{i=0}^{m}a_{j}^{i}s_{j}^{i}$ and $\bar{s} =\sum _{j=1}^{w}p_{j}\bar{s}_{j}$. This gives

94 $$\begin{aligned} \dot{p}_{j}= & {} p_{j}\left( \sum _{i=0}^{m}a_{j}^{i}\left( f_{j}^{i}+s_{j}^{i}\right) -\sum _{z=1}^{w}p_{z}\sum _{i=0}^{m}a_{z}^{i}\left( f_{z}^{i}+s_{z}^{i}\right) \right) = \end{aligned}$$

95 $$\begin{aligned}&p_{j}\left( \left( \sum _{i=0}^{m}a_{j}^{i}f_{j}^{i}-\sum _{z=1}^{w}p_{z} \sum _{i=0}^{m}a_{z}^{i}f_{z}^{i}\right) +\left( \sum _{i=0}^{m}a_{j}^{i}s_{j}^{i}-\sum _{z=1}^{w}p_{z} \sum _{i=0}^{m}a_{z}^{i}s_{z}^{i}\right) \right) .\nonumber \\ \end{aligned}$$

The equation on the scaling parameter is

96 $$\begin{aligned} \dot{n}=n\bar{r}=n\left( \sum _{j=1}^{w}p_{j}\sum _{i=0}^{m}a_{j}^{i}\left( f_{j}^{i}+s_{j}^{i}\right) -1\right) . \end{aligned}$$

Then to add neutral density dependence the fertilities $f_{j}^{i}$ should be multiplied by the logistic suppression coefficient $\left( 1-n/K\right)$ leading to the system:

97 $$\begin{aligned} \dot{a}_{j}^{i}= & {} a_{j}^{i-1}s_{j}^{i-1}-a_{j}^{i}\left( \bar{f}_{j}\left( 1-\frac{n}{K}\right) +\bar{s}_{j}\right) , \end{aligned}$$

98 $$\begin{aligned} \dot{p}_{j}= & {} p_{j}\left( \left( \bar{f}_{j}-\bar{f}\right) \left( 1-\frac{n }{K}\right) +\left( \bar{s}_{j}-\bar{s}\right) \right) , \end{aligned}$$

99 $$\begin{aligned} \dot{n}= & {} n\left( \bar{f}\left( 1-\frac{n}{K}\right) +\bar{s}-1\right) , \end{aligned}$$

giving

100 $$\begin{aligned}&\dot{a}_{j}^{i}=a_{j}^{i-1}s_{j}^{i-1}-a_{j}^{i}\left( \sum _{z=0}^{m}a_{j}^{z}f_{j}^{z}\left( 1-\frac{n}{K}\right) +\sum _{z=0}^{m}a_{j}^{z}s_{j}^{z}\right) , \end{aligned}$$

101 $$\begin{aligned}&\dot{p}_{j}=p_{j}\left( \left( \sum _{i=0}^{m}a_{j}^{i}f_{j}^{i}-\sum _{z=1}^{w}p_{z} \sum _{i=0}^{m}a_{z}^{i}f_{z}^{i}\right) \left( 1-\frac{n}{K}\right) \right. \nonumber \\&\left. \qquad +\left( \sum _{i=0}^{m}a_{j}^{i}s_{j}^{i}-\sum _{z=1}^{w}p_{z} \sum _{i=0}^{m}a_{z}^{i}s_{z}^{i}\right) \right) , \end{aligned}$$

102 $$\begin{aligned}&\dot{n}=n\left( \sum _{j=1}^{w}p_{j}\sum _{i=0}^{m}a_{j}^{i}f_{j}^{i}\left( 1- \frac{n}{K}\right) +\sum _{j=1}^{w}p_{j}\sum _{i=0}^{m}a_{j}^{i}s_{j}^{i}-1\right) . \end{aligned}$$

## Appendix D: Derivation of system $S_{b}$

System $S_{b}$ produces more complex equations. As in “Appendix C”, to add neutral density dependence the fertilities should be multiplied by the logistic suppression coefficient $\left( 1-\dfrac{n}{K}\right)$. Again we start from the Malthusian equations

103 $$\begin{aligned} \dot{n}_{j}^{0}= & {} \sum _{i=0}^{m}n_{j}^{i}f_{j}^{i}\left( 1-\dfrac{n}{K} \right) -n_{j}^{0}=n_{j}^{0}\left( \sum _{i=0}^{m}\frac{n_{j}^{i}}{n_{j}^{0}} f_{j}^{i}\left( 1-\dfrac{n}{K}\right) -1\right) , \end{aligned}$$

104 $$\begin{aligned} \dot{n}_{j}^{i}= & {} s_{j}^{i-1}n_{j}^{i-1}-n_{j}^{i}=n_{j}^{i}\left( s_{j}^{i-1}\frac{n_{j}^{i-1}}{n_{j}^{i}}-1\right) . \end{aligned}$$

Deriving the equations for the growth of age classes in the global population, in coordinates

105 $$\begin{aligned} n^{i}=\sum _{j=1}^{w}n_{j}^{i},p_{j}^{i}=\dfrac{n_{j}^{i}}{n^{i} }, \end{aligned}$$

we obtain

106 $$\begin{aligned}&\dot{n}^{0}=\sum _{j=1}^{w}\left( \sum _{i=0}^{m}n_{j}^{i}f_{j}^{i}\left( 1- \dfrac{n}{K}\right) -n_{j}^{0}\right) =\sum _{j=1}^{w}\sum _{i=0}^{m}n_{j}^{i}f_{j}^{i}\left( 1-\dfrac{n}{K}\right) -\sum _{j=1}^{w}n_{j}^{0}.\nonumber \\ \end{aligned}$$

Since $$n^{0}=\sum _{j=1}^{w}n_{j}^{0}$$ and

107 $$\begin{aligned} \sum _{j=1}^{w}\sum _{i=0}^{m}n_{j}^{i}f_{j}^{i}\left( 1-\dfrac{n}{K}\right)= & {} \sum _{i=0}^{m}n^{i}\sum _{j=1}^{w}p_{j}^{i}f_{j}^{i}\left( 1-\dfrac{n}{K} \right) = \end{aligned}$$

108 $$\begin{aligned} n^{0}\sum _{i=0}^{m}\dfrac{n^{i}}{n^{0}}\sum _{j=1}^{w}p_{j}^{i}f_{j}^{i} \left( 1-\dfrac{n}{K}\right)= & {} n^{0}\sum _{i=0}^{m}\dfrac{a^{i}}{a^{0}} \sum _{j=1}^{w}p_{j}^{i}f_{j}^{i}\left( 1-\dfrac{n}{K}\right) , \end{aligned}$$

the above equation has form:

109 $$\begin{aligned} \dot{n}^{0}=n^{0}\left( \sum _{i=0}^{m}\dfrac{a^{i}}{a^{0}} \sum _{j=1}^{w}p_{j}^{i}f_{j}^{i}\left( 1-\dfrac{n}{K}\right) -1\right) . \end{aligned}$$

Analogously we have $n^{i}=\sum _{j=1}^{w}n_{j}^{i}$ and

110 $$\begin{aligned} \dot{n}^{i}=\sum _{j=1}^{w}\left( s_{j}^{i-1}n_{j}^{i-1}-n_{j}^{i}\right) =n^{i-1}\sum _{j=1}^{w}p_{j}^{i-1}s_{j}^{i-1}-n^{i}. \end{aligned}$$

In the new coordinates the equation for the population size will be :

111 $$\begin{aligned} \dot{n}= & {} \sum _{i=0}^{m}\dot{n}^{i}=\sum _{i=0}^{m}n^{i} \sum _{j=1}^{w}p_{j}^{i}f_{j}^{i}\left( 1-\dfrac{n}{K}\right) -n^{0}+\sum _{i=1}^{m}\left( n^{i-1}\sum _{j=1}^{w}p_{j}^{i-1}s_{j}^{i-1}-n^{i}\right) \nonumber \\= & {} n\sum _{i=0}^{m}a^{i}\sum _{j=1}^{w}p_{j}^{i}f_{j}^{i}\left( 1-\dfrac{n}{K} \right) +n\sum _{i=1}^{m}a^{i-1}\sum _{j=1}^{w}p_{j}^{i-1}s_{j}^{i-1}-n, \end{aligned}$$

and since $s^{m}=0$ we can denote the above equation as

112 $$\begin{aligned} \dot{n}= & {} n\left( \bar{f}\left( 1-\dfrac{n}{K}\right) +\bar{s}-1\right) \nonumber \\= & {} n\left( \sum _{i=0}^{m}a^{i}\sum _{j=1}^{w}p_{j}^{i}f_{j}^{i}\left( 1-\dfrac{n }{K}\right) +\sum _{i=0}^{m}a^{i}\sum _{j=1}^{w}p_{j}^{i}s_{j}^{i}-1\right) . \end{aligned}$$

Therefore (109,110,112) can be presented as the replicator dynamics (11,12)

113 $$\begin{aligned}&\dot{a}^{i}=a^{i-1}\sum _{j=1}^{w}p_{j}^{i-1}s_{j}^{i-1}-a^{i} \sum _{z=0}^{m}a^{z}\left( \sum _{j=1}^{w}p_{j}^{z}f_{j}^{z}\left( 1-\dfrac{n}{ K}\right) +\sum _{j=1}^{w}p_{j}^{z}s_{j}^{z}\right) , \qquad \end{aligned}$$

114 $$\begin{aligned}&\dot{n}=n\left( \sum _{i=0}^{m}a^{i}\left( \sum _{j=1}^{w}p_{j}^{i}f_{j}^{i}\left( 1-\dfrac{n}{K}\right) +\sum _{j=1}^{w}p_{j}^{i}s_{j}^{i}\right) -1\right) . \end{aligned}$$

The above system for each age class should be completed by the replicator dynamics describing the changes of strategic composition of this particular age class. Since $a^{i}p_{j}^{i}=n_{j}^{i}/n$ we have the following form of (103):

115 $$\begin{aligned} \dot{n}_{j}^{0}= & {} n_{j}^{0}\left( \dfrac{\sum _{i=0}^{m}a^{i}p_{j}^{i}f_{j}^{i} }{a^{0}p_{j}^{0}}\left( 1-\dfrac{n}{K}\right) -1\right) \Rightarrow \end{aligned}$$

116 $$\begin{aligned} \dot{p}_{j}^{0}= & {} p_{j}^{0}\left( \dfrac{ \sum _{i=0}^{m}a^{i}p_{j}^{i}f_{j}^{i}}{a^{0}p_{j}^{0}} -\sum _{z=1}^{w}p_{z}^{0}\dfrac{\sum _{i=0}^{m}a^{i}p_{z}^{i}f_{z}^{i}}{ a^{0}p_{z}^{0}}\right) \left( 1-\dfrac{n}{K}\right) \end{aligned}$$

117 $$\begin{aligned}= & {} \frac{1}{a^{0}}\left( \sum _{i=0}^{m}a^{i}p_{j}^{i}f_{j}^{i}-p_{j}^{0}\sum _{i=0}^{m}a^{i} \sum _{z=1}^{w}p_{z}^{i}f_{z}^{i}\right) \left( 1-\dfrac{n}{K}\right) ; \end{aligned}$$

analogously for other age classes we have that (104) can be presented as

118 $$\begin{aligned} \dot{n}_{j}^{i}=n_{j}^{i}\left( s_{j}^{i-1}\frac{a^{i-1}p_{j}^{i-1}}{ a^{i}p_{j}^{i}}-1\right) , \end{aligned}$$

leading to the replicator dynamics

119 $$\begin{aligned} \dot{p}_{j}^{i}= & {} p_{j}^{i}\left( \frac{a^{i-1}p_{j}^{i-1}}{a^{i}p_{j}^{i}} s_{j}^{i-1}-\sum _{z=1}^{w}p_{z}^{i}\frac{a^{i-1}p_{z}^{i-1}}{a^{i}p_{z}^{i}} s_{z}^{i-1}\right) \end{aligned}$$

120 $$\begin{aligned}= & {} \frac{a^{i-1}}{a^{i}}\left( p_{j}^{i-1}s_{j}^{i-1}-p_{j}^{i}\sum _{z=1}^{w}p_{z}^{i-1}s_{z}^{i-1}\right) . \end{aligned}$$

Now system $S_{b}$ can be completed

121 $$\begin{aligned}&\dot{p}_{j}^{0}=\frac{1}{a^{0}}\left( \sum _{i=0}^{m}a^{i}p_{j}^{i}f_{j}^{i}-p_{j}^{0}\sum _{i=0}^{m}a^{i} \sum _{z=1}^{w}p_{z}^{i}f_{z}^{i}\right) \left( 1-\dfrac{n}{K}\right) , \end{aligned}$$

122 $$\begin{aligned}&\dot{p}_{j}^{i}=\frac{a^{i-1}}{a^{i}}\left( p_{j}^{i-1}s_{j}^{i-1}-p_{j}^{i}\sum _{z=1}^{w}p_{z}^{i-1}s_{z}^{i-1}\right) , \end{aligned}$$

123 $$\begin{aligned}&\dot{a}^{i}=a^{i-1}\sum _{j=1}^{w}p_{j}^{i-1}s_{j}^{i-1}-a^{i} \sum _{z=0}^{m}a^{z}\left( \sum _{j=1}^{w}p_{j}^{z}f_{j}^{z}\left( 1-\dfrac{n}{ K}\right) +\sum _{j=1}^{w}p_{j}^{z}s_{j}^{z}\right) , \qquad \end{aligned}$$

124 $$\begin{aligned}&\dot{n}=n\left( \sum _{i=0}^{m}a^{i}\left( \sum _{j=1}^{w}p_{j}^{i}f_{j}^{i}\left( 1-\dfrac{n}{K}\right) +\sum _{j=1}^{w}p_{j}^{i}s_{j}^{i}\right) -1\right) . \end{aligned}$$

This is the system (26,27,28,29).

## Appendix E: Rescaling the McKendrick von Foerster model to frequencies

We can rescale the McKendrick von Foerster (10) equation to relative frequencies $a(t,l)=n(t,l)/n(t)$ where $n(t)=\int _{0}^{\infty }n(t,l)dl$ is the size of the whole population. In this case the interaction rate $\tau$ should be explicitly considered. Note that in the game theoretic applications the vital rates $\tau d(t,l)$ and $\tau f(t,l)$ will change in time due to the changes of the strategic population composition. Since $n(t,l)=a(t,l)n(t)$ and the dynamics of the population size satisfies the Malthusian equation $\dfrac{dn(t)}{\partial t} =n(t)\bar{r}(t)=n(t)\tau \tilde{r}(t)$ Eq. (10) can be presented as

$$\begin{aligned} \frac{\partial a(t,l)}{\partial t}n(t)+\frac{\partial n(t)}{\partial t}a(t,l)+\frac{ \partial a(t,l)}{\partial l}n(t)=-\tau d(t,l)a(t,l)n(t). \end{aligned}$$

After substitution $\dfrac{\partial n(t)}{\partial t}=n(t)\tau \tilde{r}(t)$ and division by *n*(*t*) we obtain

125 $$\begin{aligned} \frac{\partial a(t,l)}{\partial t}+\frac{\partial a(t,l)}{\partial l}=a(t,l)\left[ -\tau d(t,l)-\tau \tilde{r}(t)\right] , \end{aligned}$$

where per capita growth rate

$$\begin{aligned}&\tau \tilde{r}(t)=\tau \bar{f}(t)\left( 1-\dfrac{n(t)}{K}\right) -\tau \bar{d}(t)= \\&\tau \left[ \left( 1-\dfrac{n(t)}{K}\right) \int _{0}^{\infty }a(t,l)f(l)dl-\int _{0}^{\infty }n(t,l)d(l)dl\right] \end{aligned}$$

and the boundary condition will be

$$\begin{aligned} a(t,0)=n(t,0)/n(t)=\tau \left( 1-\frac{n(t)}{K}\right) \int _{0}^{\infty }a(t,l)f(t,l)dl. \end{aligned}$$

Note that we can remove $\tau$ from Eq. (125) by a change of timescale. We can use Eq. (125) as the PDE equivalent of Eq. (20) from system $S_{a}$ and (28) from $S_{b}$, and rescale Eq. (10) for *j*th strategy to $$p_{j}(t,l)=n_{j}(t,l)/n(t,l)\,\ $$(where $n(t,l)=\sum _{j}n_{j}(t,l)$) to obtain the PDE equivalent of Eqs. (26,27). Then since $n_{j}(t,l)=p_{j}(t,l)n(t,l)$,

$$\begin{aligned}&\frac{\partial p_{j}(t,l)}{\partial t}n(t,l)+\frac{\partial n(t,l)}{\partial t} p_{j}(t,l)+\frac{\partial p_{j}(t,l)}{\partial l}n(t,l)+\frac{\partial n(t,l)}{ \partial l}p_{j}(t,l)\\&\quad =-\tau d_{j}(t,l)p_{j}(t,l)n(t,l), \end{aligned}$$

which leads to

$$\begin{aligned}&\left[ \frac{\partial p_{j}(t,l)}{\partial t}+\frac{\partial p_{j}(t,l)}{\partial l} \right] n(t,l)+\left[ \frac{\partial n(t,l)}{\partial t}+\frac{\partial n(t,l)}{ \partial l}\right] p_{j}(t,l)\\&\quad =-\tau d_{j}(t,l)p_{j}(t,l)n(t,l). \end{aligned}$$

Substituting using Eq. (10) describing whole population and division by *n*(*t*, *l*) leads to:

$$\begin{aligned} \frac{\partial p_{j}(t,l)}{\partial t}+\frac{\partial p_{j}(t,l)}{\partial l}=-\tau p_{j}(t,l)\left[ d_{j}(t,l)-\bar{d}(t,l)\right] , \end{aligned}$$

and the boundary condition replacing Eq. (26) is $p_{j}(t,0)=n_{j}(t,0)/n(t,0)=\tau \left( 1-\frac{n}{K}\right) \int _{0}^{\infty }p_{j}(t,l)f_{j}(t,l)dl$. Parameter $\tau$ can easily be removed in the resulting equivalents of systems $S_{a}$ and $S_{b}$ by the timescale adjustment, thus the continuous framework is timescale independent and can be driven by the game demographic payoff functions.

## Appendix F: Derivation of the payoff functions for the age structured sex ratio model

Our operational payoff functions of active individuals (41) and ( 43) can be presented in new coordinates in the following way:

126 $$\begin{aligned} f_{m}^{op}(P_{j},a,G,M)= & {} \dfrac{k}{2}\left( \dfrac{x}{y}\bar{P}_{pr}+ \dfrac{x_{j}}{y_{j}}P_{j}\right) , \end{aligned}$$

127 $$\begin{aligned}= & {} \dfrac{k}{2}\left( \dfrac{1-P_{op}}{P_{op}}\bar{P}_{pr}+\dfrac{ 1-M_{j}^{op}}{M_{j}^{op}}P_{j}\right) , \end{aligned}$$

128 $$\begin{aligned} f_{f}^{op}(P_{j},a,G,M)= & {} \dfrac{k}{2}\left( \left( 1-P_{j}\right) +\dfrac{ y_{j}}{x_{j}}\left( 1-\bar{P}_{pr}\right) \dfrac{x}{y}\right) , \end{aligned}$$

129 $$\begin{aligned}= & {} \dfrac{k}{2}\left( \left( 1-P_{j}\right) +\dfrac{M_{j}^{op}}{1-M_{j}^{op}} \left( 1-\bar{P}_{pr}\right) \dfrac{1-P_{op}}{P_{op}}\right) . \end{aligned}$$

Note that in the age structured case *x*, *y*, $x_{j}$ and $y_{j}$ describe the numbers of sexually active individuals of both sexes. Males are active in age classes from *a* to *b* and females from *c* to *d*. Fractions of sexually active females and males in the new formulation can be presented in the form:

130 $$\begin{aligned} S_{j}^{f}=\sum _{i=c}^{d}a_{j}^{i}(1-M_{j}^{i})\text { and }S_{j}^{m}=\sum _{i=a}^{b}a_{j}^{i}M_{j}^{i}. \end{aligned}$$

Then $\bar{S}^{f}=\sum _{j=1}^{w}G_{j}S_{j}^{f}$ and$\bar{S} ^{m}=\sum _{j=1}^{w}G_{j}S_{j}^{m}$ are the respective averages, and operational sex ratios are $M_{j}^{op}=\dfrac{S_{j}^{m}}{S_{j}^{m}+S_{j}^{f}}$and $P_{op}=\dfrac{\bar{S}^{m}}{\bar{S}^{m}+\bar{S}^{f}}$. Then the operational fertility payoff function of a gene carrier will be

131 $$\begin{aligned} f_{g}^{op}(P_{j},a,G,M)= & {} M_{j}^{op}f_{m}^{op}(P_{j},a,G,M)+\left( 1-M_{j}^{op}\right) f_{f}^{op}(P_{j},a,G,M) \nonumber \\= & {} \dfrac{k}{2}\left( M_{j}^{op}\left( \dfrac{1-P_{op}}{P_{op}}\bar{P}_{pr}+ \dfrac{1-M_{j}^{op}}{M_{j}^{op}}P_{j}\right) \right. \nonumber \\&\quad \left. +\left( 1-M_{j}^{op}\right) \left( \left( 1-P_{j}\right) +\dfrac{M_{j}^{op}}{1-M_{j}^{op}}\left( 1-\bar{P }_{pr}\right) \dfrac{1-P_{op}}{P_{op}}\right) \right) \nonumber \\= & {} \dfrac{k}{2}\left( M_{j}^{op}\dfrac{1-P_{op}}{P_{op}}\bar{P}_{pr}+\left( 1-M_{j}^{op}\right) P_{j}\right. \nonumber \\&\quad \left. +\left( 1-M_{j}^{op}\right) \left( 1-P_{j}\right) +M_{j}^{op}\left( 1-\bar{P}_{pr}\right) \dfrac{1-P_{op}}{P_{op}}\right) \nonumber \\= & {} \dfrac{k}{2}\left( \left( 1-M_{j}^{op}\right) +M_{j}^{op}\dfrac{1-P_{op}}{ P_{op}}\right) . \end{aligned}$$

To obtain the per capita gene carrier fertility payoff, necessary to derive the replicator equations, we multiply the above by the fraction of active carriers

132 $$\begin{aligned} \left[ S_{j}^{m}+S_{j}^{f}\right] =\left[ \sum _{i=a}^{b}a_{j}^{i}M_{j}^{i}+ \sum _{i=c}^{d}a_{j}^{i}\left( 1-M_{j}^{i}\right) \right] \end{aligned}$$

leading to

133 $$\begin{aligned} f_{g}(P_{j},a,G,M)= & {} \dfrac{k}{2}\left( \left( 1-M_{j}^{op}\right) +M_{j}^{op}\dfrac{1-P_{op}}{P_{op}}\right) \left[ S_{j}^{m}+S_{j}^{f}\right] \end{aligned}$$

134 $$\begin{aligned}= & {} \dfrac{k}{2}\left( S_{j}^{f}+S_{j}^{m}\dfrac{1-P_{op}}{P_{op}}\right) \end{aligned}$$

135 $$\begin{aligned}= & {} \dfrac{k}{2}\left( S_{j}^{f}+S_{j}^{m}\dfrac{\bar{S}^{f}}{\bar{S}^{m}} \right) . \end{aligned}$$

Similarly we derive the per capita average fertility. Then the average payoff (48) becomes the operational average fertility of the active individuals

136 $$\begin{aligned} f^{op}(a,G,M)=k\left( 1-P_{op}\right) . \end{aligned}$$

Again to obtain the per capita average fertility payoff we multiply this by the fraction of active individuals in the population $\sum _{j=1}^{w}G_{j} \left[ S_{j}^{m}+S_{j}^{f}\right] =\bar{S}^{m}+\bar{S}^{f}$. This leads to

137 $$\begin{aligned} \bar{f}(a,G,M)=k\left( 1-P_{op}\right) \left( \bar{S}^{m}+\bar{S}^{f}\right) =k\bar{S}^{f}. \end{aligned}$$

## Appendix G: Derivation of the age structured replicator equations

The bracket describing the fertility stage of the gene pool dynamics will be

138 $$\begin{aligned}&\left( f_{g}(P_{j},a,G,M)-\bar{f}(a,G,M)\right) =\dfrac{k}{2}\left( S_{j}^{f}+S_{j}^{m}\dfrac{1-P_{op}}{P_{op}}\right) -k\bar{S}^{f}= \end{aligned}$$

139 $$\begin{aligned}&\dfrac{k}{2}\left[ S_{j}^{f}+S_{j}^{m}\dfrac{\bar{S}^{f}}{\bar{S}^{m}}-2\bar{ S}^{f}\right] =\dfrac{k}{2}\left[ S_{j}^{f}+\left[ \dfrac{S_{j}^{m}}{\bar{S} ^{m}}-2\right] \bar{S}^{f}\right] = \end{aligned}$$

140 $$\begin{aligned}&k\left[ \dfrac{1}{2}\left( \dfrac{S_{j}^{f}}{\bar{S}^{f}}+\dfrac{S_{j}^{m}}{ \bar{S}^{m}}\right) -1\right] \bar{S}^{f}. \end{aligned}$$

The bracket describing the mortality stage will be

141 $$\begin{aligned}&\left( \bar{s}_{j}-\bar{s}\right) =\left( \sum _{i=0}^{m}a_{j}^{i}\bar{s} _{j}^{i}-\sum _{z=1}^{w}G_{z}\sum _{i=0}^{m}a_{z}^{i}\bar{s}_{z}^{i}\right) = \end{aligned}$$

142 $$\begin{aligned}&\left( \sum _{i=0}^{m}a_{j}^{i}\left[ M_{j}^{i}s_{m}^{i}+\left( 1-M_{j}^{i}\right) s_{f}^{i}\right] -\sum _{z=1}^{w}G_{z} \sum _{i=0}^{m}a_{z}^{i}\left[ M_{z}^{i}s_{m}^{i}+\left( 1-M_{z}^{i}\right) s_{f}^{i}\right] \right) ,\nonumber \\ \end{aligned}$$

since $\bar{s}_{j}^{i}=M_{j}^{i}s_{m}^{i}+\left( 1-M_{j}^{i}\right) s_{f}^{i}$ is the average survival of the *j*th strategy carrier in age class *i*. Futhermore

143 $$\begin{aligned} \bar{s}_{j}=\sum _{i=0}^{m}a_{j}^{i}\left[ M_{j}^{i}s_{m}^{i}+\left( 1-M_{j}^{i}\right) s_{f}^{i}\right] \text { and }\bar{s} =\sum _{j=1}^{w}G_{j}\bar{s}_{j} \end{aligned}$$

are the average $P_{j}$ carrier and population survival probabilities, leading to

144 $$\begin{aligned} \dot{G}_{j}=G_{j}\left( k\left[ \dfrac{1}{2}\left( \dfrac{S_{j}^{f}}{\bar{S} ^{f}}+\dfrac{S_{j}^{m}}{\bar{S}^{m}}\right) -1\right] \bar{S}^{f}\left( 1- \dfrac{n}{K}\right) +\left( \bar{s}_{j}-\bar{s}\right) \right) \end{aligned}$$

which is Eq. (65). Now we derive the equations describing the dynamics of sex ratios in the particular age classes in the subpopulation of carriers of strategy $P_{j}$ which will be equivalent to the original equations on $M_{j}$. We use the version of Eq. (26) for two types (sexes) where the same payoffs are obtained in certain age ranges specific for each type (thus the term $\sum _{i=0}^{m}a^{i}p_{j}^{i}f_{j}^{i}$ will be $f_{1}\sum _{i=a}^{b}a^{i}p_{1}^{i}$). In effect (26) for type 1 (males) reduces to

145 $$\begin{aligned} \dot{p}_{1}^{0}=\dfrac{f_{1}\sum _{i=a}^{b}a^{i}p_{1}^{i}-p_{1}^{0}\bar{f}}{ a^{0}}\left( 1-\dfrac{n}{K}\right) , \end{aligned}$$

since $\bar{f}=\sum _{i=0}^{m}a^{i}\sum _{z=1}^{w}p_{z}^{i}f_{z}^{i}$ in (26) is the average fertility in the population (in our case the subpopulation of carriers of the *j*-th strategy). To translate the above equation into the notation used in the sex ratio model we should apply the following substations: $p_{1}^{0}\rightarrow M_{j}^{0}$, $f_{1}\rightarrow f_{m}^{op}$, $f_{2}\rightarrow f_{f}^{op}$ and $\bar{f}\rightarrow f_{g}$ since $\sum _{i=a}^{b}a^{i}p_{1}^{i}$ is equivalent to $S_{j}^{m}$. Here there are no different strategies indexed by a lower index but two opposite sexes, thus for any particular gene we will have a single equation describing the sex ratio in the zero age class:

146 $$\begin{aligned} \dot{M}_{j}^{0}=\dfrac{\left( f_{m}^{op}(P_{j},a,G,M)S_{j}^{m}-M_{j}^{0}f_{g}(P_{j},a,G,M)\right) }{ a_{j}^{0}}\left( 1-\dfrac{n}{K}\right) . \end{aligned}$$

Recalling (56) that $\left( 1-M_{j}^{op}\right) /M_{j}^{op}=S_{j}^{f}/S_{j}^{m}$ and $\left( 1-P_{op}\right) /P_{op}=\bar{S} ^{f}/\bar{S}^{m}$ leads to Eq. (67),

147 $$\begin{aligned} \dot{M}_{j}^{0}= & {} \dfrac{\left( \dfrac{k}{2}\left( \dfrac{1-P_{op}}{P_{op}} \bar{P}_{pr}+\dfrac{1-M_{j}^{op}}{M_{j}^{op}}P_{j}\right) S_{j}^{m}-M_{j}^{0} \dfrac{k}{2}\left( S_{j}^{f}+S_{j}^{m}\dfrac{1-P_{op}}{P_{op}}\right) \right) }{a_{j}^{0}}\nonumber \\&\left( 1-\dfrac{n}{K}\right) \nonumber \\= & {} \dfrac{k}{2a_{j}^{0}}\left( S_{j}^{m}\dfrac{\bar{S}^{f}}{\bar{S}^{m}}\bar{P} _{pr}+S_{j}^{m}\dfrac{S_{j}^{f}}{S_{j}^{m}} P_{j}-M_{j}^{0}S_{j}^{f}-M_{j}^{0}S_{j}^{m}\dfrac{\bar{S}^{f}}{\bar{S}^{m}} \right) \left( 1-\dfrac{n}{K}\right) \nonumber \\= & {} \dfrac{k}{2a_{j}^{0}}\left( S_{j}^{m}\left( \bar{P}_{pr}-M_{j}^{0}\right) \dfrac{\bar{S}^{f}}{\bar{S}^{m}}+S_{j}^{f}\left( P_{j}-M_{j}^{0}\right) \right) \left( 1-\dfrac{n}{K}\right) . \end{aligned}$$

Note that the above equation is equivalent to the Tug of War dynamics of the original model, and so should be completed by the respective equations for all age classes (27), which are Eq. (68),

148 $$\begin{aligned} \dot{M}_{j}^{i}=\dfrac{a_{j}^{i-1}}{a_{j}^{i}}\left( M_{j}^{i-1}s_{m}^{i-1}-M_{j}^{i}\bar{s}_{j}^{i-1}\right) . \end{aligned}$$

The equations describing the age structure (28) of the entire population of carriers of the *j*-th strategy (with $f_{g}$ acting as $\bar{f }_{j}$) will be Eq. (66)

149 $$\begin{aligned}&\dot{a}_{j}^{i}=a_{j}^{i-1}\bar{s}_{j}^{i-1}-a_{j}^{i}\left[ f_{g}(P_{j},a,G,M)\left( 1-\dfrac{n}{K}\right) +\bar{s}_{j}\right] = \nonumber \\&a_{j}^{i-1}\left[ M_{j}^{i-1}s_{m}^{i-1}+\left( 1-M_{j}^{i-1}\right) s_{f}^{i-1}\right] -a_{j}^{i}\left[ \dfrac{k}{2}\left( S_{j}^{f}+S_{j}^{m} \dfrac{\bar{S}^{f}}{\bar{S}^{m}}\right) \left( 1-\dfrac{n}{K}\right) +\bar{s} _{j}\right] = \nonumber \\&a_{j}^{i-1}\bar{s}_{j}^{i-1}-a_{j}^{i}\left[ \dfrac{k}{2}\left( S_{j}^{f}+S_{j}^{m}\dfrac{\bar{S}^{f}}{\bar{S}^{m}}\right) \left( 1-\dfrac{n }{K}\right) +\bar{s}_{j}\right] . \end{aligned}$$

From (64) and (59) we obtain the population size Eq. ( 69). Therefore we have derived the system (65,66,67,68,69).
